# Dihydrostreptomycin Directly Binds to, Modulates, and Passes through the MscL Channel Pore

**DOI:** 10.1371/journal.pbio.1002473

**Published:** 2016-06-09

**Authors:** Robin Wray, Irene Iscla, Ya Gao, Hua Li, Junmei Wang, Paul Blount

**Affiliations:** 1 Department of Physiology, University of Texas Southwestern Medical Center, Dallas, Texas, United States of America; 2 School of Pharmacy, Tongji Medical College, Huazhong University of Science and Technology, Wuhan, China; 3 School of Traditional Chinese Materia Medica, Shenyang Pharmaceutical University, Shenyang, China; 4 Green Center for Systems Biology and Biophysics, University of Texas Southwestern Medical Center, Dallas, Texas, United States of America; Oxford University, UNITED KINGDOM

## Abstract

The primary mechanism of action of the antibiotic dihydrostreptomycin is binding to and modifying the function of the bacterial ribosome, thus leading to decreased and aberrant translation of proteins; however, the routes by which it enters the bacterial cell are largely unknown. The mechanosensitive channel of large conductance, MscL, is found in the vast majority of bacterial species, where it serves as an emergency release valve rescuing the cell from sudden decreases in external osmolarity. While it is known that MscL expression increases the potency of dihydrostreptomycin, it has remained unclear if this effect is due to a direct interaction. Here, we use a combination of genetic screening, MD simulations, and biochemical and mutational approaches to determine if dihydrostreptomycin directly interacts with MscL. Our data strongly suggest that dihydrostreptomycin binds to a specific site on MscL and modifies its conformation, thus allowing the passage of K^+^ and glutamate out of, and dihydrostreptomycin into, the cell.

## Abstract 2

The antibiotic dihydrostreptomycin binds to a specific site on the bacterial mechanosensitive channel MscL, opens the pore, and appears to pass through to access the cytoplasm of the cell.

## Author Summary

Streptomycin is one of the original and best studied antibiotics. Its primary mechanism of action is the interference with protein synthesis by binding to and modifying the function of the bacterial ribosome. However, the antibiotic is quite large, bulky, and is charged, so the mechanisms by which it accesses the inside of the bacterial cell have remained largely unknown. Previously, we have found that the expression of a bacterial mechanosensitive channel, MscL, increases the potency of a variant of this antibiotic, dihydrostreptomycin. Here, we define the dihydrostreptomycin binding site on the MscL channel. We also show how this antibiotic modifies the channel and opens the pore, allowing the diffusion of solutes, such as potassium and glutamate, from the cytoplasm of the cell out to the medium. Finally, we provide evidence that dihydrostreptomycin can pass through MscL and that the channel is thus one pathway by which the drug accesses the inside of the bacterial cell.

## Introduction

Streptomycin is a member of the aminoglycoside family of antibiotics, with bactericidal properties. The mechanism of action has been extensively studied since its discovery in 1944. Ultimately, it binds to the S12 protein of the 30S subunit on the ribosome and inhibits translation and can cause misreading of mRNA [1]. However, it is not clear how streptomycin, or its more potent derivative, dihydrostreptomycin (DHS), gets into the cell. With a diameter of about 10 Å and a net charge of 3^+^, DHS is not an obvious candidate for easily crossing a biological membrane. Interestingly, upon treatment with the drug, an outward flux of K^+^ from the cell has been observed prior to any decrease in cell viability [2], suggesting either the opening of a channel or some sort of loss of cell membrane integrity.

**Fig 1:**
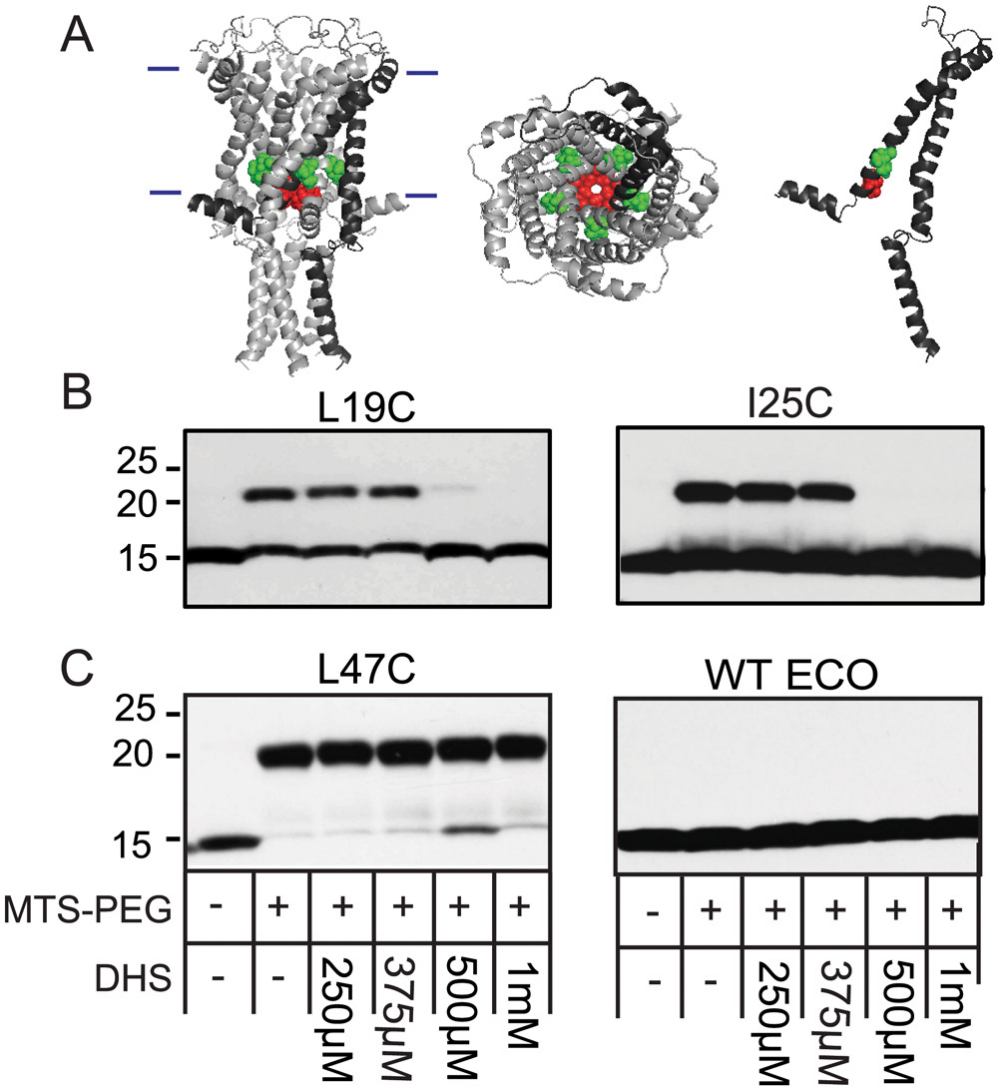
DHS blocks the binding of MTS-PEG5000 to cysteine at L19C and I25C in a dose-dependent manner. *(A)* Ec-MscL structure from molecular modeling, based on the Mt-MscL crystal structure, with the molecules viewed from the lipid bilayer (left). The approximate location of the bilayer is indicated with horizontal bars. A single subunit is darkened for clarity. The view from the periplasmic side (middle) and a single subunit (right) are also shown. The location of the L19 residue is shown in red, I25 is shown in green. *(B)* Western blot analysis after MTS-PEG5000 versus DHS competition assay. The absence (−) or presence (+) of 50 μM MTS-PEG5000 (MTS-PEG), as well as the absence (−) or different concentration of DHS used, are indicated in the table at the bottom. Note that the upper band is protein that has been PEGylated; this band disappears as the concentration of DHS is increased (500 μM–1 mM). *(C)* Shown are negative controls for this assay: an unrelated Ec-MscL with a mutation in the periplasm, L47C, shows a PEGylated band that is not inhibited or shifted by DHS (left), and WT Ec-MscL, which does not have any naturally occurring cysteine, shows no upper band with MTS-PEG5000 (right).

MscL is a bacterial mechanosensitive channel of large conductance that responds directly to tension in the membrane [3–6]. It serves as a biological “emergency release valve” that allows a rapid loss of solutes and osmoprotectants, including K^+^ and glutamate, in response to a sudden decrease in osmolality in the bacterial environment, thus protecting the cell from lysis [7]. Under very special conditions and mutagenesis, small ions have also been shown to be able to pass through the channel into the cytoplasm [7,8]. The crystal structure for the MscL homolog from *Mycobacterium tuberculosis* (Mt-MscL) has been solved by the Rees group revealing a homopentameric channel with each subunit having two transmembrane domains [9,10]. The first transmembrane domain (TM1) forms the pore and is linked to the second transmembrane domain (TM2), which is in contact with the lipid bilayer, via a periplasmic loop that is not as conserved as the rest of the protein. The N-terminal domain is a helix that runs parallel to the cytoplasmic membrane; the C-terminal forms a cytoplasmic helical bundle (Fig 1A). Biochemical, structural-functional, and molecular dynamics (MD) studies have all been used to study the channel [5,6]. In sum, much of the data has suggested that TM1 must undergo a large clockwise (>100°) rotation with both transmembranes tilting into the lipid bilayer to open the large 30 Å pore [11] in the channel. The inappropriate opening of such a large pore would be predicted to have negative physiological consequences; indeed, genetic analysis has shown that mutations in key areas of the MscL protein, including the pore, can lead to a MscL channel that gates more easily and, when expressed in vivo, is detrimental to bacterial growth [12,13].

In a previous study, we used a high-throughput screen in search of compounds that would target the *Escherichia coli* MscL (Ec-MscL), increase its activity, and thus inhibit bacterial growth [14]. Surprisingly, one of the positive hits was DHS. Subsequent studies demonstrated that Ec-MscL, as well as MscL orthologues from *Bacillus subtilis* and *Staphylococcus aureus*, when expressed in *E*. *coli*, increased the potency of DHS relative to cells that were null for the protein. In addition, we found that MscL expression was required for the DHS-dependent K^+^ flux observed prior to decreased cell viability. However, from the data, it remained unclear if MscL directly or indirectly influenced MscL function, if DHS uses the large MscL pore as a pathway to enter the cell, and if DHS can modify the MscL channel in streptomycin-resistant cells.

MD simulations of biomolecules provide a detailed view of structure and dynamics that complement experiments. Increased conformational sampling, enabled by new algorithms and growth in computer power, [15,16] now allows a much broader range of events to be observed, providing critical insights, largely inaccessible to experiments. MD simulation of MscL has been used to investigate how the lipid composition affects the structures and dynamics of Mt-MscL, particularly the charge-rich segment RKKEEP located several amino acid distal to the end of TM2 [17]. The gating mechanisms have been extensively studied by MD simulations for both Mt-MscL [18,19] and Ec-MscL [20]. All of the MD studies suggest that the interplay between membrane and protein plays a key role in the opening and closing events of the MscL channel. However, because of a lack of specific agonists and antagonists, MD simulations have not before been used to determine how small biomolecules influence the MscL structure or determine if or how they can pass through the MscL channel.

Here, we use a combination of genetic screening, biochemical and mutational approaches, computational analyses, MD simulations, and in vivo assays to determine if DHS’ effects on the MscL channel are direct. Our data strongly suggest that DHS directly binds to a specific location in the channel pore and modifies the channel conformation in such a way as to allow the passage of K^+^ and glutamate out of, and DHS into, the cell.

## Results

### A Cysteine Library Screen Reveals a Possible Binding Area for DHS

Under the hypothesis that mutations in MscL that disrupt DHS binding to the channel could lead to increased bacterial growth in the presence of DHS, we designed a 96-well plate screen utilizing a cysteine-scan library previously described [21,22]. Briefly, we screened mutations S2C-E107C, omitting strong gain of function (GOF) mutations because of their slowed growth phenotype, and loss of function (LOF) mutations because of their inability to gate upon normal stimuli. The concentration of DHS used, 6.25 μM, was optimized to be just subthreshold for bacteria expressing the wild type Ec-MscL in order to enhance the sensitivity of the assay. At this concentration, growth of cultures expressing the wild type MscL channel was inhibited, on average, by 50%. Positive results were defined as yielding at least a 50% increased growth over cultures expressing wild type MscL in three independent experiments. Only two cysteine mutants were consistently positive by these criteria in all three experiments: L19C and I25C. When highlighted on a model for the structure of the Ec-MscL [23], which is based on the Mt-MscL crystal structure [10], these residues lie in the constriction point of the pore of the channel (Fig 1A). Although these residues are not adjacent, one must appreciate that different residues can be exposed because of the twisting of TM1 upon channel opening [24], and asymmetry has been predicted by several experiments in the opening of the channel [19,25,26]; thus, these residues may contribute to not just a single but perhaps an evolving binding pocket for a large chemical such as DHS. Previous studies have demonstrated that mutations and post-translational changes in this region can influence channel sensitivity to membrane tension; hydrophilic substitutions and charges within this region make the channel easier to gate [13,21]. Hence, it is conceivable that the multicharged (3^+^) DHS, when bound within the pore, could also increase MscL sensitivity.

### MTS-PEG5000 Competition Experiments Support the Cysteine Library Screen Results

Although the screening data, above, are suggestive that these residues participate in DHS binding and mode of action, we sought additional biochemical support. Because cysteine residues can react with sulfhydryl reagents, they can be modified by methanosulfonate (MTS) reagents. MTS-PEG5000 potentially gives us the ability to have site-directed PEGylation of any of our cysteine library residues [27]. If the cysteine mutation lies within the DHS binding site but does not fundamentally alter it, then high concentrations of this antibiotic should compete for MTS-PEG5000 binding. Because of its large size, the PEGylated proteins can then be separated from non-PEGylated ones by SDS-PAGE; any potential inhibition of this PEG modification by DHS competition can easily be assessed. Thus, we performed such an experiment on purified L19C and I25C Ec-MscL mutated channels. As seen in Fig 1B, in both instances, DHS at concentrations greater than 500 μM appeared to compete with MTS-PEG5000. In contrast, a MscL channel with a cysteine mutation in the periplasmic loop region of the protein, L47C, an area that is not expected to interact with DHS, still showed a gel shift due to PEGylation, but this shift was not inhibited by DHS even at high concentrations (1 mM). (Fig 1C). Finally, wild type MscL, which contains no endogenous cysteines, showed no apparent increase of mass attributed to PEGylation (Fig 1C). This competition was specific to DHS since another aminoglycoside antibiotic, spectinomycin, at concentrations up to 10 mM showed no decrease in the PEGylation band (S1 Fig). Thus, it appears that higher concentrations of DHS, but not spectinomycin, can block the binding of MTS-PEG5000 to the cysteines located at L19 and I25 within the Ec-MscL.

### Variation between Ec-MscL, a MscL Orthologue, and Appropriate Substitutions in Each, Further Support the Hypothesis That L19 Contributes to DHS Binding and Mechanism of Action

**Fig 2:**
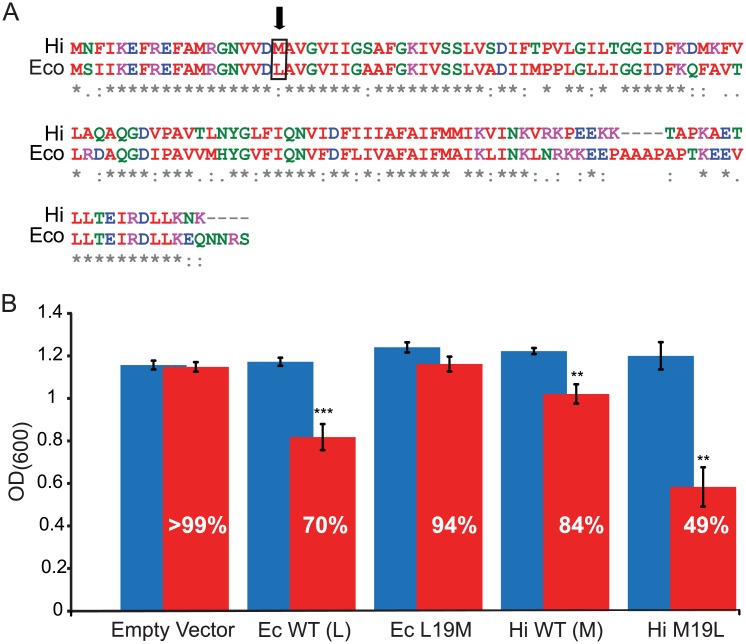
Mutational analysis provides additional evidence that MscL L19 contributes to the binding site of, and increased sensitivity to, DHS. *(A)* Sequence alignment of two MscL homologs from *E*. *coli* (Ec) and *H*. *influenzae* (Hi) showing the difference in sequence at amino acid 19 (boxed and arrow). *(B)* Average OD_600_ and SEM from six independent experiments are shown for both WT and mutations of *E*. *coli* MscL (Ec) *and H*. *influenzae* MscL (Hi) after 240 min in the presence (red bars) or absence (blue bars) of 6.25 μM DHS. The percentage of growth in the presence of DHS, relative to the no DHS, is shown. Note that changing the Ec-MscL L19 to M decreases DHS sensitivity, while changing Hi-MscL M19 to L increases sensitivity. *n = 6*, **p < .*005*, ***p <. 0005 versus no DHS added 2-tailed paired *t* test.

If L19 and I25 contribute to DHS binding and mode of action, then one might expect that variations at these sites between orthologues could lead to differences in DHS sensitivity. Both of these residues are highly conserved. In assessing the cases in which differences in residues are seen at these sites, we noted that for the analogous L19 site, the vast majority of orthologues contained the canonical L, with only a small minority containing an M at this location. The *Haemophilus influenzae* orthologue (Hi-MscL), which has been functionally characterized [28], was one orthologue within this minority. *H*. *influenzae* is, like *E*. *coli*, gram-negative, and Hi-MscL is very homologous to the Ec-MscL channel (Fig 2A). Growth for Ec-MscL, Hi-MscL, and mutated channels was measured in the presence and absence of 6.25 μM DHS (Fig 2B). Interestingly, while cells expressing Ec-MscL, which contains the L at position 19, showed a 30% decrease in growth due to DHS, cells expressing wild type Hi-MscL, which has an M at this position, showed only a 16% decrease. If L19 truly contributes to DHS binding and action, then mutating it to an M in Ec-MscL should decrease the DHS sensitivity of cells expressing this mutated channel. Indeed, L19M Ec-MscL expressing cells showed only a 6% decrease in growth in the presence of DHS. Even more significant was the observation that when M19 of Hi-MscL is changed to L, a “super sensitive” channel is formed; cells expressing this mutated channel show a substantial 51% decrease in cell growth.

One trivial explanation for these data could be that the orthologues and mutated channels had different sensitivities to membrane tension and thus were more or less likely to be modified by DHS. Hence, these mutated and WT Hi-MscL channels were assayed by both patch clamp and in an in vivo hypo-osmotic down shock assay to assess their function. If the higher sensitivity of Ec-MscL to DHS was due to increased channel sensitivity to membrane tension, then one would expect it to be more sensitive than Hi-MscL; however, the opposite was observed in patch clamp, and no differences were noted between the wild type channels and their respective mutants (S2A and S2B Fig). In addition, all channels were shown to be functional in vivo (S2C Fig), increasing the viability of osmotically fragile cells from osmotic lysis (survival close to 80%, although at 74%, the L19M Ec-MscL-expressing cells were close to, but statistically less than, the 80% for wild-type Ec-MscL; LOF mutants are usually defined as a 50% decrease in viability compared to wild type [22].

Presumably, these data translate to the concentration at which DHS modifies the channel. To more directly test this, we used MicroScale Thermophoresis (MST) [29,30]. In initial experiments using this approach, we found the affinity for the wild type Ec-MscL was relatively weak (Kd = 9.81 mM). On the assumption that large conformational changes are affecting the signal, and wild-type MscL may be somewhat resistant to such large changes when solubilized, we used the more sensitive mutant K55T, which has a sensitivity identical to that of the Hi-MscL M19L mutant (in these mechanosensitivity experiments, MscS is used as an internal standard and thresholds measured [31,32]; MscL/MscS threshold ratios of 1.11 ± 0.05 where *n* = 5 and 1.10 ± 0.04, where *n* = 10 were determined for Ec-MscL K55T and Hi-MscL M19L, respectively). Indeed, using MTS, the K55T Ec-MscL gave a better apparent affinity, relative to the wild type channel, of 680 μM. Note that this binding affinity to DHS, which was performed on solubilized protein, is in the approximate range of those observed in our MTS-PEG5000 inhibition assays, which have also been performed on solubilized channels. Consistent with our interpretation that lower concentrations of DHS effect significant changes on the M19L Hi-MscL, this channel gave an even better apparent affinity of 50 μM (S3 Fig). As expected, no affinity could be measured for the unrelated compound isoniazid, which was used as a negative control.

Together, these data derived from the Hi- and Ec-MscL orthologues, and complementary mutations, strongly support the notion that L19 contributes to DHS binding and modulation of the MscL protein.

### MD Simulations of Ec-MscL

To determine the conformational changes in MscL that may occur upon interactions with DHS, we performed MD simulations. The starting structure for all MD simulations is a homology model based on the crystal structure of Mt-MscL resolved at 3.5 Å. The details on homology modeling, system setup, molecular docking, and MD simulation protocols are provided in the Materials and Methods. First, 240 nanoseconds MD simulation was carried out for the homology model in a simulation box consisting of 230 POPC lipids, 95 KCl and 32368 water molecules. The overall root-mean-square deviations (RMSDs) of α-carbon, about 3.6 Å compared to the crystal structure, are quite reasonable (see Materials and Methods). The last snapshot of the MD trajectory was selected as a representative conformation for the following DHS molecular docking study.

**Fig 3:**
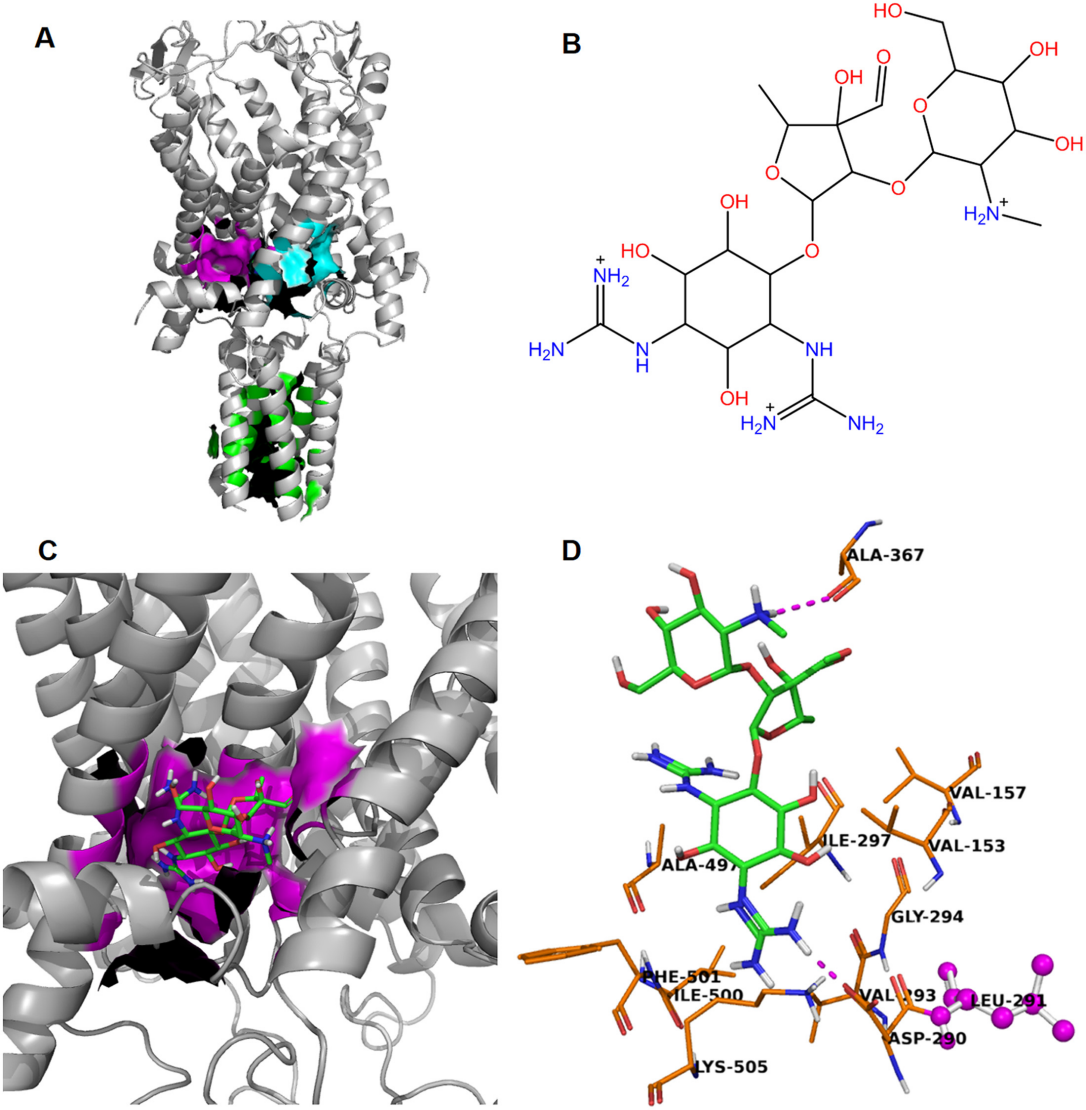
Docking studies representing how DHS binds to the closed conformation of Ec-MscL. (A) Three binding sites identified by SiteID using the last snapshot of the ligand-free MD simulation. The green binding site is located inside of the channel formed by 19 C-terminal helical residues from monomer 1: L122, I125, L129, Q132, N133; monomer 2: L258 (L122), L265 (L129); monomer 3: L401 (L129), Q404 (Q132), N405 (N133); monomer 4: L530 (L122), I533 (I125), L536 (L128), Q540 (Q132); monomer 5: L666 (L122), I669 (I125), L672 (L128), L673 (L129), Q676(Q132)). If a binding site residue does not belong to the first monomer, then the corresponding residue ID of the first monomer is provided in the followed parenthesis. The magenta binding site has a smooth deep pocket formed by residues from monomer 2: V153 (V17), V157 (V21); monomer 3: D290 (D18), V293 (V21), G294 (G22), I297 (I25); monomer 4: A497 (A89), I500 (I92), F501 (F93), and K505 (K97). The cyan binding pocket is the smallest in term of volume of the cavity and formed by Residues from monomer 1: F10; monomer 2: D154 (D18), G158 (G22), I161 (I25); monomer 3: A361 (A89), I364 (I92), F365 (F93), I368 (I96), and K369 (K97). (B) Molecular structure of DHS. (C) The best docking pose of DHS bound to the magenta binding site. (D) A focused look at DHS and its surrounding residues at the magenta binding site. Two hydrogen bonds (magenta dashed-line) are formed between the ammonium functional group of DHS and A367 (A95) and one guanidinium functional group of DHS with monomer 3: D290 (D18). L291 (L19), shown in magenta ball and stick representation, is known as a “hot spot” in the MTSPEG5000 competition assay.

The first step of a molecular docking study is to identify possible binding sites. Three possible binding sites were discovered by the SiteID module of SYBYL [33] and shown in Fig 3A. GLIDE (Grid-based Ligand Docking with Energetics) [34] docking was then performed for DHS (Fig 3B) at each binding site. The best docking score, −7.46 kcal/mol, was achieved for the magenta pocket formed by ten amino acid residues (Fig 3C). No meaningful docking pose was discovered at the cyan and green binding pockets.

As shown in Fig 3D, two hydrogen bonds are formed between DHS and the surrounding residues. One guanidinium functional group of DHS forms a salt bridge with monomer 3: D290 (D18). Also, monomer 3: I297 (I25) is a magenta binding pocket residue, and monomer 3 L291 (L19), in magenta ball and stick representation, has a very close contact with DHS and several binding pockets residues, even though itself is not recognized by SiteID as binding pocket residue. These latter findings are consistent with the experimental finding that DHS is a competitor of binding MTSPEG5000 to cysteine at L19C and I25C.

We previously speculated that DHS could pass through the MscL channel if the channel partially or fully opened [14]. We therefore tested if DHS could be seen to pass through the channel using MD simulations. First, DHS was manually placed in the center of the Ec-MscL pore with ten different orientations. For each docking orientation, 50 nanosecond MD simulation was first performed with the secondary structure domains (TM1, TM2, S1 and C-terminal helix) and lipids strongly restrained followed by another 100 nanosecond regular MD simulation without any restraint.

**Fig 4:**
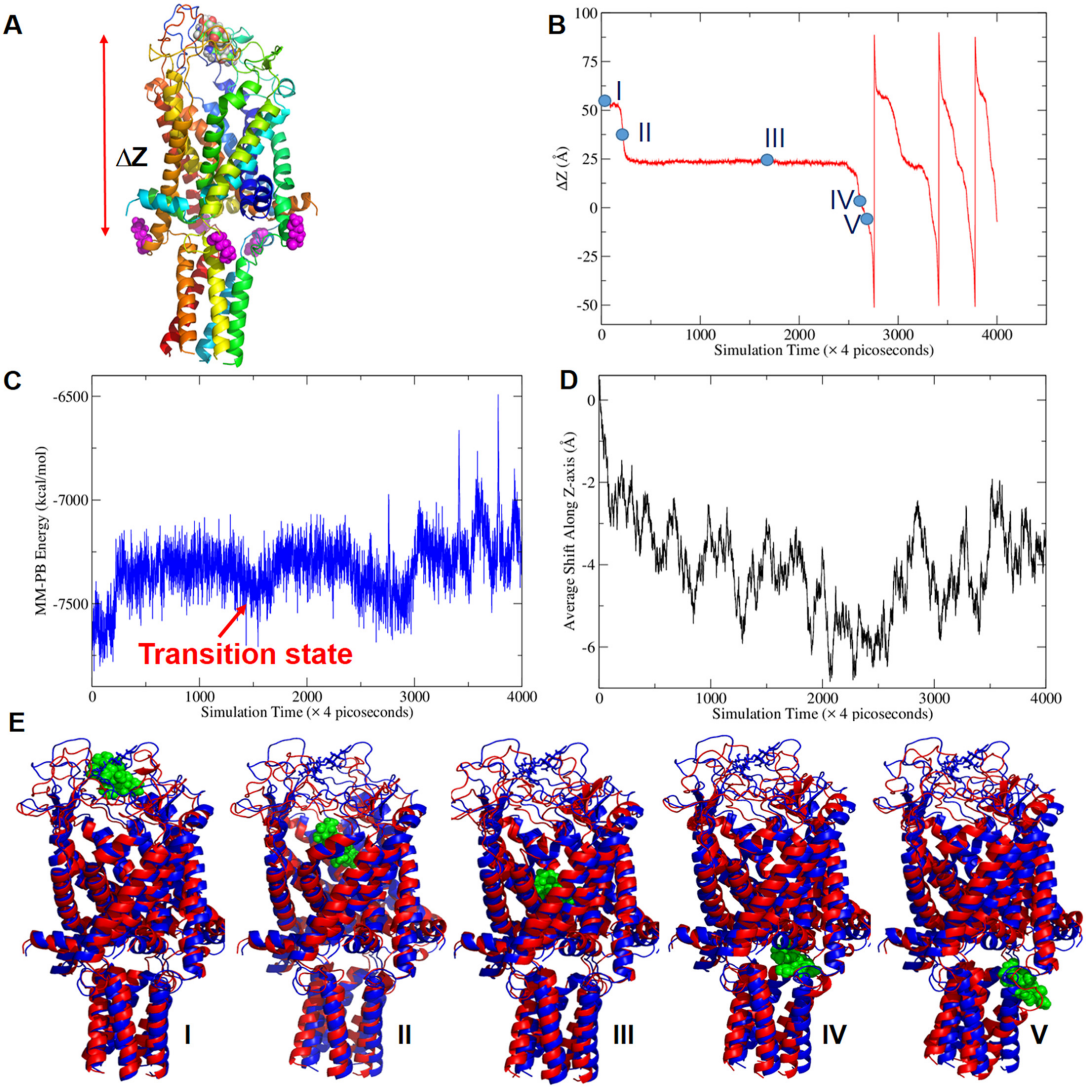
Molecular dynamic simulation of DHS passing through the Ec-MscL channel with an EEF of 0.2 volt/Å applied to DHS. (A) ΔZ is defined as the Z coordinate difference of two geometrical centers of DHS and five K106 LYS residues (monomer 1: K106; monomer 2: K242; monomer 3: K378; monomer 4: K514, and monomer 5: K650) which are K106 in monomer labeling. If DHS is located in or above the plane formed by the five LYS, ΔZ > = 0, otherwise ΔZ < 0. (B) The ΔZ plot of DHS passing through Ec-MscL. Five representative conformations labeled as I, II, III, IV, and V, were selected for structural analysis. (C) The fluctuation of the MM-PB energy of Ec-MscL/DHS along the MD simulation trajectory of the passing-through event. (D) The average shifts along Z-axis of 95 K^+^ during the MD simulation. The negative displacements imply ions go towards outside of the membrane. (E) Representative conformations of the five stages of DHS passing through the Ec-MscL channel event superposed to the last snapshot of the ligand-free MD simulation. DHS is shown as green balls. The reference structure in blue representation is a closed conformation. All the representative conformations of the five passing-through stages are shown as red ribbons. From Conformations I to V, the C-terminal helixes and TM1 are deformed progressively, while no significant difference is observed for S1 and TM2.

In order to observe the passages of DHS through the Ec-MscL channel within a short timeframe for MD simulations, an external electric field (EEF) was applied to DHS to accelerate the process. Indeed, a previous study has found that increased membrane potentials in vivo increase the potency of DHS [35]. Note that the experiment was designed so that only the DHS molecule felt this field. Only the best docking orientation, which was recognized according to both the global and ensemble conformational energies sampled by the 100-nanosecond MD simulations, was selected for the subsequent MD simulations with EEF (Fig 4A). With the EEF strength being set to 0.2 volt/Å along the Z-axis, we observed the complete procedure of the passing-through event within 12 nanoseconds (Fig 4B). These data have been repeated a total of five times (twice where the Cl^-^ molecules were set to balance the positive charges), all with consistent results with the passage of DHS through the MscL channel. The modified pore is more than accommodating for DHS, which does not have to dehydrate for passage; in fact, DHS is surrounded by tens to hundreds of water molecules when it passes through the channel. A video of the passing-through event is provided as S1 Video.

We defined a parameter, ΔZ, the Z-coordinate difference of two geometric centers formed by DHS and five LYS residues located at the end of TM2 (Fig 4), to describe the passing through event. As demonstrated in Fig 4B, DHS passed through Ec-EscL four times, and the last three took a much shorter time since the channel had already changed conformation. It is also clear that there is a long time period (about 8 nanoseconds) that DHS stayed in an intermediate state with a characteristic ΔZ value of 25 Å.

We further characterized the passing-through event by calculating the MM-PB energies of all the collected MD snapshots. As shown in Fig 4C, the intermediate state has favorable MM-PB energies consistent with the fact that DHS stays in the middle of the channel for about half of the time of the simulation. The methods and computational details on PB calculations are presented in the Materials and Methods. Moreover, we calculated the displacements of K^+^ during the passing-through event. The average displacements along Z-axis of 95 K^+^, about 6 Å towards the outside of the membrane was observed (Fig 4D). This means that the channel is more open and, although there is no electric field on the ion, it begins to migrate in the direction it would need to flow for efflux from the cell. This finding is consistent with the observation of K^+^ efflux in in vivo experiments [2,14].

The whole procedure can be roughly divided into five stages according to the residence time and the MM-PB energies: (I) initial stage (0.72 nanoseconds), (II) fast transition stage (0.4 nanoseconds), (III) transition stage (8.8 nanoseconds), (IV) pre-exiting stage (0.76 nanoseconds), and (V) exiting stage (0.36 nanoseconds). The representative conformations of the five stages are shown in Fig 4E. From Stage I to V, DHS moved from the MscL pore to the cytoplasmic side of the membrane progressively. How each secondary structure units (transmembrane domains TM1 and TM2, N-terminal S1 and C-terminal helix) fluctuate along the MD simulation trajectory is discussed further in the Materials and Methods. Interestingly, the RMSDs of the two transmembrane domains and S1 increased slowly from the initial stage to the intermediate state stage and then the final stages; on the contrary, the C-terminal domain has a dramatic RMSDs surge at the final stages (Stages VI and V) to accommodate DHS exiting from the channel.

The RMSDs of the five representative conformations are summarized in S1 Table. Moreover, we also quantitatively characterized the helical rotation during the MscL channel gating induced by the DHS passing-through event. The axis angles calculated from rotational matrices of least-squares fittings were listed in the S2 Table for the five representative conformations; they are divided by the S1 helices (S2A Table), TM1 helices (S2B Table), TM2 helices (S2C Table), and C-terminal helices (S2D Table). Compared to the closed conformation, the first TM1 helix rotates in a corkscrew manner (clockwise as seen from the periplasm) from 10.5° in Stage I to 16.3° in Stage III and 27.5° in Stage V. Large movements also were observed for the C-terminal helical bundle, which largely moved as a unit: the rotational axis angles increased about 10 to 15° for four of the five helices from Stage I to Stage V, although the final exiting stage contributes the most changes.

**Fig 5:**
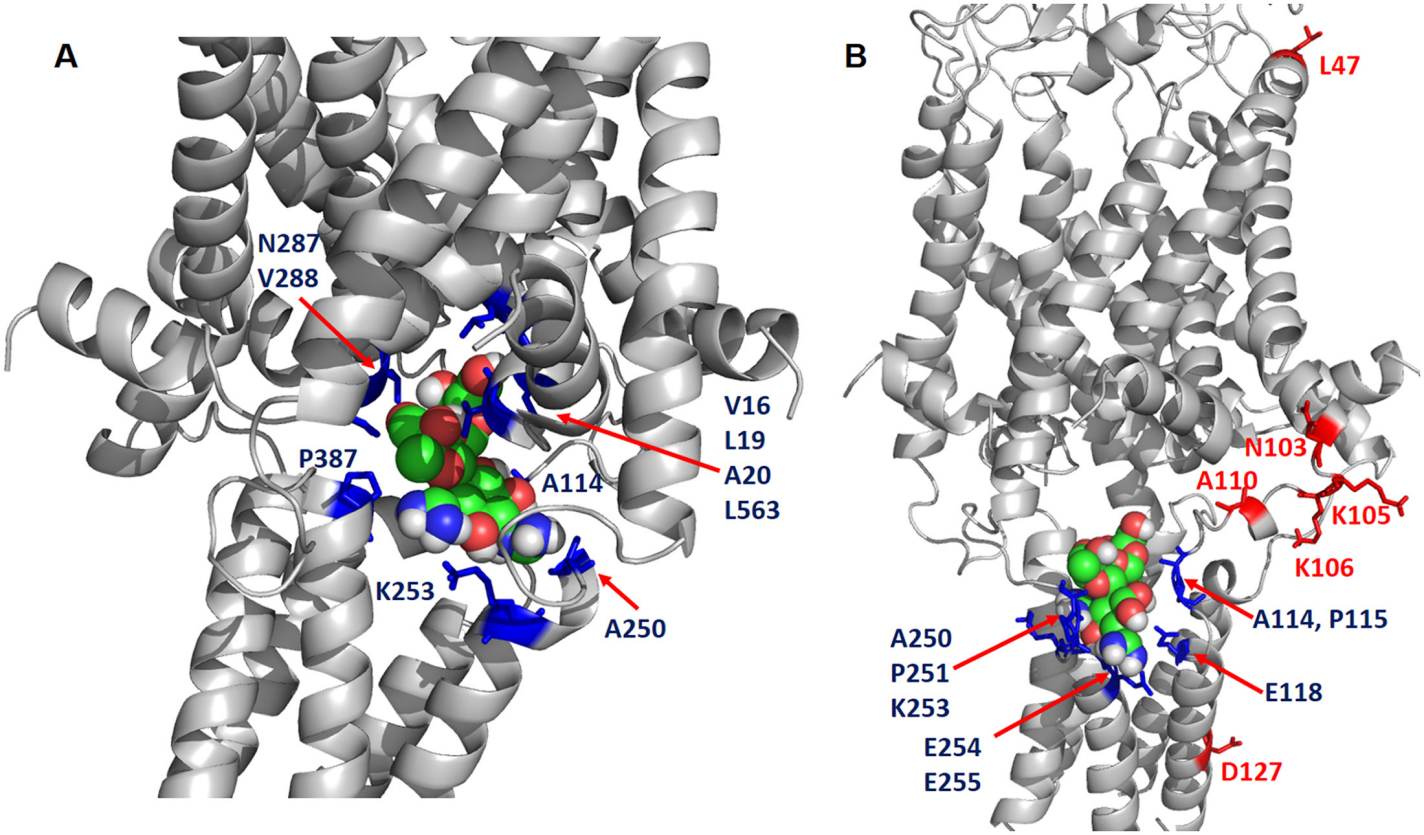
Identification of “hot spots” that are critical for the Ec-MscL/DHS binding using the representative conformations in the last two stages. DHS is represented by spheres, the “hot spots” shown as blue sticks have strong interactions with DHS, while red stick are predicted negative in the competition assay since they are all resided on the surface of Ec-MscL. (A) Stage IV: The residue IDs of “hot spots” include monomer 1: V16, L19, A20, A114; monomer 2: A250 (A114), K253 (K117); monomer 3: N287 (N15), V288 (V16), P387 (P115) and monomer 4: L563 (L155). (B) Stage V. The residue IDs of “hot spots” include monomer 1: A114, P115, E118; monomer 2: A250 (A114), P251 (P115), K253 (K117), E254 (E118), and E255 (E119). The red residues which are used as control in the competition assay include L47, N103, A110, K105, K106 and D127. Note that the residue IDs in parentheses are the corresponding residue IDs in Ec-MscL monomer.

Key residues that directly interact with DHS were identified for representative conformations of the five stages (Fig 5, S4 and S5 Figs). The conformations of DHS in the five stages are similar, particularly for Stages I-III. DHS adopts a prolonged conformation to facilitate its exit from the channel in Stages IV and V (S6 Fig). We then characterized the MscL channel by calculating the pore radii along the channel coordinate. The “W” shape plot in S7 Fig suggests that there are two bottlenecks (around -50 and -10 Å) and the latter is narrower and needs more time for opening. This result is consistent with our MD result (Fig 4B). This plot also suggests that the passage of small molecule like DHS does not lead to a fully open Ec-MscL channel.

### MD Snapshots Predict Additional Sites for DHS Binding and Interactions

**Fig 6:**
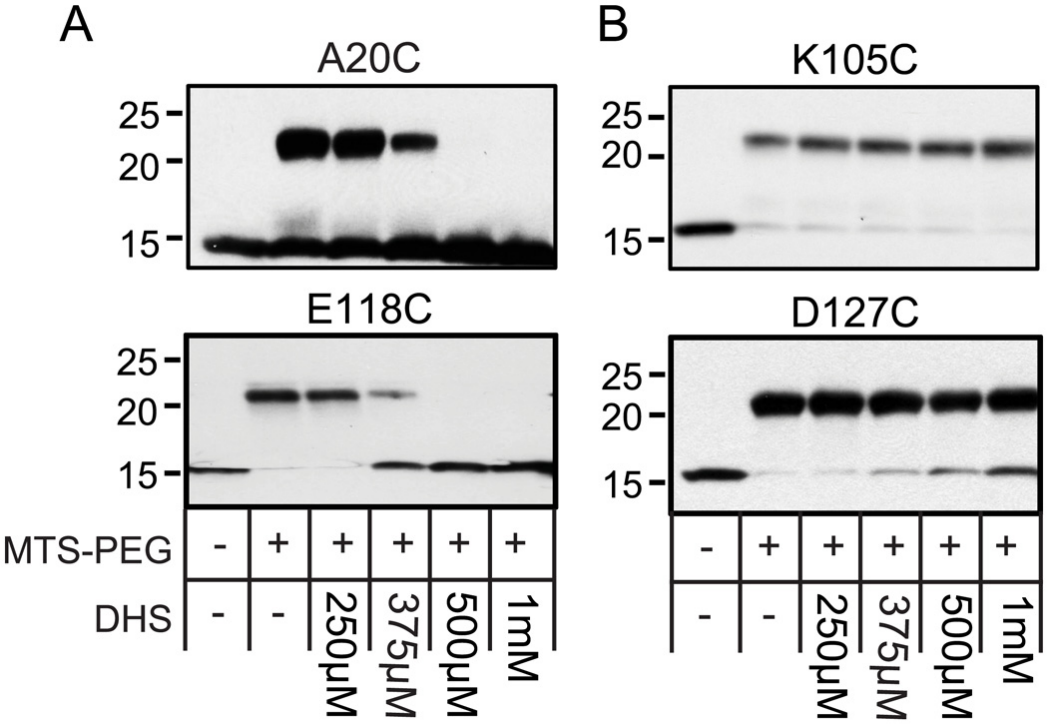
MD simulation snapshots predict additional residues that interact with DHS. *(A)* western blot analysis after a competition experiment of two residues predicted to interact with DHS, using the cysteine mutations indicated. The absence (−) or presence (+) of 50 μM MTS-PEG5000 (MTS-PEG), as well as the absence (−) or different concentration of DHS used, are indicated in the table at the bottom. Note that the upper band is protein that has been PEGylated, and that this band disappears as the concentration of DHS is increased (500 μM–1 mM). *(B)* Western blot analysis after a competition experiment of two residues predicted not to interact with the antibiotic, using the cysteine mutations indicated. Upper PEGylated bands can be seen for all concentrations of DHS (250 μm to1 mM).

The MD simulation experiments have made predictions of points of interaction between DHS and the MscL protein. Using both the magenta binding site from SiteID as well as several representative snapshots selected from the MD simulation of DHS passing through Ec-MscL channel, ten residues in MscL were predicted to participate in this interaction: V16, V17, D18C, L19, A20, A114, P115, T116, K117, and E118 (Fig 5). We therefore subjected these cysteine-mutated residues to the MTS-PEG5000 competition assay described above. We chose two residues reflecting the two distinct domains proposed to interact with DHS (A20C, and E118C) As shown in Fig 6A, each is positive, as predicted, suggesting they are indeed involved in DHS binding and interactions; the remaining eight residues were also found to be positive and are shown in S8 Fig.

In addition, we have tested five residues in the area predicted NOT to interact with DHS, N103, K105, K106, A110, and D127. In Fig 6B, we show two of the more distant residues, K105C and D127C, and neither showed inhibition of the PEGylation in the presence of DHS. Shown in S8 Fig are the additional mutated channels; K106C and A110C were negative as predicted, but N103C was positive. This latter finding may be explained by a previously defined conformational change of the protein that makes this site inaccessible as the channel opens; we found that this residue becomes buried within the lipid bilayer upon gating [36]. The evidence that N103 becomes buried within lipids includes phenotypes, including channel sensitivities, obtained after residue modifications designed to embed the residue in the membrane or stabilize it at the membrane interface. In addition, the residue was mutated to tryptophan, and the amount of fluorescence quenching by brominated lipids was measured upon channel gating. All of the data supported a model in which this site becomes buried within the lipids in an early transition state as the channel opens. Assuming this to be the case, it is therefore not surprising that the site is not PEGylated when in the presence of DHS; the DHS is opening the channel and thus burying the 103 site and preventing modification. Given this background, we decided to evaluate the contact numbers for N103 interacting with lipid hydrophobic sites within the MD simulations. The average contact numbers for N103 are 2.52, 4.03, 3.38, 3.30, and 2.42 for the five conformational stages defined in Fig 4. In contrast, the average contact number is only 0.01 for the closed conformation. It is noted that the fast transition stage and the transition state stage have the largest and the second largest contact numbers, and the contact numbers decrease gradually once DHS begins passing through the channel. This MD result suggests that N103 can regulate Ec-MscL gating by increasing or decreasing the lipid exposure; these data are entirely consistent with previous experimental data [36]. It therefore seems likely that upon interacting with DHS, the channel undergoes a conformational change that buries this residue within a hydrophobic pocket making it inaccessible for MTS-PEG5000 modification.

### DHS Modifies the MscL Channel Even in DHS-Resistant Strains: MscL-Dependent Glutamate Fluxes in DHS-Resistant Cell Lines

We previously demonstrated that MscL expression changes the potency of DHS [14]. The finding that increasing the concentration of DHS still leads to decreased viability in sensitive cells suggested MscL is not the only pathway into the cytoplasm, thus explaining why the *mscL* gene has never been identified in streptomycin suppressor assays. In addition, there is no obvious slowed-growth or decreased-viability phenotype for DHS-resistant strains when in the presence of DHS, demonstrating, not surprisingly, that MscL is not the primary mode of action of the drug. However, we have previously shown that upon treatment with DHS, there is a MscL-dependent flux of both K^+^ and glutamate in bacteria that were sensitive to streptomycin [14], strongly suggesting that the DHS-dependent flux of these osmolytes is due to MscL activation. This begs the question: can we measure subtler MscL- and DHS-dependent physiological phenotypes in DHS-resistant cells using flux assays? Thus far, we have not seen a K^+^ flux in DHS-resistant *E*. *coli* strains. There may be two explanations for this. First, there may be other factors playing a role in the perceived K^+^ flux; indeed, one interpretation of a previous study was that misfolded and truncated transmembrane proteins may intercalate into the bilayer and compromise membrane integrity [37]. It is possible that this compromising of the membrane is not direct, but in fact the aberrant proteins add tension in the membrane and increase MscL sensitivity to DHS. Under this scenario, MscL-dependent fluxes would only be seen in DHS-sensitive cells. However, a second explanation is that the many high-affinity K^+^ pumps largely compensate for fluxes in DHS-resistant cells, which are presumably healthier than DHS-sensitive cells upon DHS treatment. Note that these two explanations are not mutually exclusive and both may play a role. To avoid the latter complication of compensatory mechanisms, we assayed for glutamate, which is accumulated largely through synthesis rather than being pumped into the cell by high-affinity pumps; i.e., fluxed glutamate should not be rapidly taken back into the cell. We used two independent DHS-resistant cell lines from different parental strains that are MscL deficient: MJF367 and PB104 from Frag 1 and AW405 parental strains, respectively. As expected, upon DHS treatment there was no significant loss of glutamate from cells expressing empty plasmid in either of the cell lines (98.1% of the glutamate remained in the cells for MJF367 and 99.2% for PB104’s; *t* test *p*-values >0.1 and *n* = 3 for both). However, the amount of glutamate remaining in the MJF367 cell line expressing MscL was 78.5%, and PB104’s was 83.7%. Both of these values, while not as great as the 52% loss seen in streptomycin-sensitive cells [14], were significantly different from empty plasmid (*p*-values <0.005 for MJF367 and < .05 for PB104’s; *n* = 3 for each). Viability experiments were also performed in conjunction with the glutamate experiments, and no significant loss in viability was seen for any strain under any condition. Thus, a modest but statistically significant DHS- and MscL-dependent glutamate flux can be measured in DHS-resistant cells independent of a decreased viability, strongly suggesting that DHS can influence MscL gating independent of DHS toxicity.

Together, the data support the direct binding of DHS to a specific site within the MscL channel and its ability to modify MscL such that DHS can pass through the channel into the cytoplasm of the cell. These changes apparently occur in both streptomycin-sensitive as well as streptomycin-resistant cells but may be amplified in the latter due to a previously suggested increase of abnormal membrane proteins adding tension in the membrane and thus increasing the sensitivity of MscL.

## Discussion

Early in the study of the MscL channel, a forward genetics study found that mutations in the channel could lead to cell slowed growth or even death [13]. Briefly, the channel was randomly mutagenized, placed under transcriptional control of the inducible LacUV5 promoter, plated, then replica plated onto plates containing IPTG, which induced expression. Colonies that grew less or failed to grow on the IPTG-containing plates were further studied. In sum, the data demonstrated that mutations at several locations, including what is now known to be the pore constriction site, led to channels that were more sensitive to membrane tension as assayed by patch clamp and, when expressed, lead to the decreased growth or viability observed.

On the assumption that small chemical compounds may also be able to make the channel more sensitive, and thus may lead to the development of channel agonists and potential antimicrobial agents, we developed a high-throughput screen [14]. Because MscL senses membrane tension, any compound that intercalates into the membrane could lead to a more sensitive channel; indeed, amphipaths have previously been shown to gate mechanosensitive channels including MscL [38,39]. Hence, in the secondary screen we tested the compounds on cells expressing only *E*. *coli* MscS, a member of an unrelated second family of bacterial tension-sensing mechanosensitive channels [7]. Surprisingly, four known antibiotics were identified in the screen: DHS, spectinomycin, viomycin, and nifuroxazide. Of the four, DHS appeared to be the most specific and potent and therefore further characterized. We found that MscL expression was required for the DHS-dependent K^+^ efflux observed prior to a decrease in cell viability. Further, we demonstrated that a DHS-dependent flux of another, larger osmoprotectant fluxed by MscL, glutamate, was also observed on the same time scale and also found to be MscL dependent; here, we demonstrate that some glutamate flux can even be measured in DHS-resistant cells in a MscL-dependent manner. Moreover, MscL channel activity increased in response to DHS as assayed by patch clamp [14]. This latter finding was originally generated using the mutated channel used in the initial screen; not surprisingly, this finding holds true for the wild type *E*. *coli* MscL channel using an electrode back-fill approach [31,40], as shown in S9 Fig. However, obvious questions remained: Was DHS directly binding to and modifying the MscL channel? If so, where is this binding site located? And is MscL one conduit for passage of the large bulky DHS molecule into the cell cytoplasm? Here, we present several lines of evidence that DHS binds to the MscL pore, including an in vivo screen of over 90 mutated channels, a MTS-PEG5000 competition assay, analysis of an orthologue with variability within the predicted binding site (including mutagenesis of the site), and MD simulations. The latter showed DHS modifying the channel and passing through it. Finally, the MD simulations made predictions of residues that interact with DHS upon this modification and passage; these predictions were tested by the MTS-PEG5000 competition assay and shown to be true. The most likely binding site using the channel-closed conformation (predicted by SideID, which is in magenta in Fig 3C) is relatively stable during DHS passing through the Ec-MscL channel (S10 Fig) as well as the binding cavity volumes predicted by the SiteID software. How DHS binding to this site affects the passage of DHS through the channel is an interesting topic and will be pursued in future study.

Current models for the open structure of the channel suggest that TM1 corkscrews early in the gating process about 110° in the membrane in a clockwise direction when viewed from the periplasm, then the transmembranes tilt in the membrane and separate like the iris of a camera, opening a large pore approximately 30Å in diameter [5,6]. Many of the initial structural changes have been supported by recent crystal structures of the archaeal MscL homolog from *Methanosarcina acetivorans* trapped in both the closed and closed-expanded intermediate states [41]. The MD simulations of MscL in the presence of DHS do not show full channel opening. Perhaps it is the short time scale of the simulations, but it may also be that additional in vivo environmental factors including ambient membrane tension resulting from the predicted high cell turgor [42], or the electric field, predicted to be about −140 mV across the bacterial membrane [43,44], play a role in the sensitivity of the channel to DHS modulation. Regardless, many of the changes observed in our simulations are consistent with an opening channel. For example, the corkscrewing (up to 27.5°) of TM1 is observed in the simulations, as well as a separation of the helices around the pore/vestibule. Potassium, an osmoprotectant that normally can flux through the open channel, even though not under an electrochemical gradient, is observed to move into the channel vestibule; this is also consistent with the DHS-dependent K^+^ flux observed prior to decreased cell viability [2,14]. The observation that the C-terminal helical bundle moves as a unit, remains intact, and the DHS passes asymmetrically through the linkers between TM2 and the C-terminal helical bundle is also expected, because several studies suggest that the bundle is quite stable [45,46] and remains intact upon gating [47]. Finally, the cytoplasmic residue N103 has been predicted to insert into, then out of, the membrane in a piston-like fashion upon gating [36]; this is also observed in the MD simulations and is a likely explanation of why this residue gives an apparently false positive in the MTS-PEG5000 competition assay. In sum, although a full opening of the MscL channel is not observed, many of the conformational changes observed are consistent with the initial movements toward a fully open channel.

The finding that DHS binds the pore vestibule is, in hindsight, not surprising. The random mutagenesis study discussed above found that many mutations within this region leads to increased channel sensitivity [13]. In addition, it appeared that increasing hydrophilicity or adding charges in the pore/vestibule domain leads to inappropriate channel activity and compromised cell growth [24]. These findings, in conjunction with the crystal structure of the Mt-MscL, led us to propose the “hydrophobic lock” hypothesis, which states that it is the transient exposure of hydrophobic residues within the pore to an aqueous environment that is the major energy barrier for channel opening [48]. Interestingly, DHS interactions appear to be at the interface of two subunits within the pore vestibule. This is reminiscent of the acetylcholine binding site of the nicotinic acetylcholine receptor [49,50]; presumably, changes in protein—protein interactions at subunit interface induced by ligand binding can more easily lead to changes in global conformation, and thus channel gating. Indeed, part of the MscL binding pocket is predicted to contain residues from a neighboring TM2 domain. We have previously studied this region of the protein, both the S1 domain [51], as well as the cytoplasmic portion of TM2 [36,52,53], and even the changes in interactions that occur between these domains upon channel gating [54]. These previous studies have found this region to be highly dynamic and undergo large conformational changes upon gating. In sum, the data indicate that DHS binds to and modifies what could be referred to as the gate of the channel, i.e., the subunit interface at the most constricted portion of the pore, thus leading to a conformational change that allows the efflux of osmoprotectants and the passage of DHS into the cytoplasm.

## Materials and Methods

### Strains and Cell Growth

#### Library screen/in vivo hypo-osmotic down shock assays/Growth inhibition experiments

*E*. *coli* strain MJF455 (ΔmscL::Cm, ΔmscS) [7] was used to host the pb10 expression constructs. Cells were grown in citrate-phosphate-defined media (CphM) pH 7.0, consisting of (per liter: 8.57 g of Na_2_HPO_4_, 0.87 g of K_2_HPO_4_, 1.34 g of citric acid, 1.0 g NH_4_SO_4_, 0.001 g of thiamine, 0.1 g of Mg_2_SO_4_.7H_2_O, 0.002 g of (NH_4_)_2_SO_4_.FeSO_4_.6H_2_o), and ampicillin (100 μg/ml).

#### Protein expression

Two systems were used for protein expression. *E*. *coli* strain PB104 (Δ*mscL*::Cm) was used to host the pb10 or *E*. *coli* strain PB116 (Δ*mscL*::Cm; DE3 Lysogen) [55] was the host for pET21a expression constructs. Overnight cultures were diluted 1:100 into three litters of Lennox broth (LB) medium (Fisher Scientific) plus ampicillin (100 μg/ml) in a shaker at 37°C, rotated at 250 cycles/min. Expression was induced at an OD_600_ of 0.7–0.8 with the addition of 1 mM IPTG for 3 h at 37°C. Cultures were then pelleted at 4,000 rpm for 20 min and stored at −80°C until needed.

### Molecular Techniques

Mutations for this study were generated using the mega primer technique as previously described [22]. His 6 tags were added to existing mutants on the C-terminus with standard PCR technique. The resulting PCR products were ligated into either pB10d [51] or pET21a (Novagen).

### In Vivo Assays

#### Glutamate assay

The MJF367 (*ΔmscL*::*Cm*) [7] and PB104 (*ΔmscL*::*Cm*) [31] strains were used for the glutamate flux experiments carrying pb10d empty vector or expressing WT MscL. Overnight cultures were grown from a single colony in K10 media consisting of 46 mM Na_2_HPO_4_, 23 mM NaH_2_PO_4_, 8 mM (NH_4_)_2_SO_4_, 10 mM KCl, and 100 mM NaCl, supplemented with 0.4 mM MgSo_4_, 3 μM thiamine, 6 μM iron, 0.04% glucose plus 100 μg ml^-1^ ampicillin. The next morning cultures were diluted 1:50 in the same media; when they reached an OD_600_ = 0.2, they were induced for 1 h at 37°C with 1 mM IPTG. The cultures were then split in two: mock (no drug added) or with 60 ug/ml DHS and incubated for 20 min at 37°C. The final OD_600_ was recorded, and 15 ml of each sample were pelleted, the supernatant removed, and immediately frozen down to −80°C. The glutamate assay was developed by bringing samples up in the same media-adjusting volume for final OD_600_. Glutamate measurements were performed using the EnzyChrom Glutamate Assay Kit (BioAssay Systems, Hayward, CA, US) according to the manufacturer’s instructions. Viability assays were done in conjunction with the assay. After the final OD_600_ was taken, cultures were diluted 1:20 in the same media and vortexed. Samples were then diluted further with six consecutive 1:10 serial and 5 ul plated on LB amp plates. The following day, colony forming units were calculated.

#### Cysteine library screen

Frozen stocks of the MJF455 strain carrying MscL single cysteine mutations (S2C-E107C) were made in a 96-well plate format (GOF and LOF mutations were omitted). This plate was used to inoculate overnight cultures grown in a flat bottomed 96-well plate covered with a sterile breathable film (Axygen) in CphM at 37°C at 170 rpm. OD_595_ was taken the next morning to ensure all samples grew. 96-well plates containing 190 μl of prewarmed CphM, 1 mM IPTG or CphM, 1 mM IPTG, and DHS at 6.25 μm were inoculated with 10 ul from the overnight culture plate. Plates were sealed with breathable film, wrapped in foil and placed in a 37°C incubator without shaking for 16 h. OD_595_ was then taken, and the blank was subtracted. The difference in OD_600_ was calculated between the plate with DHS and without DHS. Hits were then selected by at least 4 times more growth compared to WT Ec-MscL.

#### Growth inhibition experiments

Cultures, in CphM, were started from a single colony with constructs in the MJF455 strain. After overnight growth, they were diluted 1:50 into the same media, CphM, and grown at 37°C, rotated at 250 rpm, to an OD_600_ of 0.2. Protein expression was then induced with 1mM IPTG. After 45 min of induction, the culture was split in two prewarmed flasks, one with no drug and one with 6.25 μm DHS. OD_600_ was taken at 240 min of growth and averaged for all experiments.

#### In vivo hypo-osmotic down shock assay

A single colony was picked for each construct in the MJF455 strain [7] and grown overnight at 37°C in CphM plus 100 ug/ml ampicillin. The overnight cultures were then diluted 1:20 in this defined medium, grown for 1 h, and then diluted to an OD_600_ of 0.05 in the same medium supplemented with 1 M NaCl for a final concentration of 0.5 M NaCl. After at least two divisions, expression was induced for 45 min with 1 mM IPTG. The induced cultures were diluted 1:20 into (i) CphM media containing 0.5 M NaCl (mock shock); (ii) water (osmotic downshock). Cells were then incubated at 37°C, without shaking, for 15 min, and then six consecutive 1:10 serial dilutions were made in medium containing either no salt (for the osmotic downshock conditions) or 0.5 M NaCl (for the mock-shock conditions). These diluted cultures were plated and grown overnight, and the colony-forming units were counted and averaged per experiment.

### Biochemical Techniques

#### Protein purification

Frozen cell pellets that expressed the His-tagged MscL cysteine mutants were lysed in 50 mM KPi pH 7.5, 150 mM NaCl, 0.5 μg/ml DNAse, 1 mg/ml lysozyme, and a complete mini EDTA-free protease tab (Roche) per 10 ml, using a French press at 16K PSI at 4°C. The resulting lysate was incubated for 1 h at room temperature (RT) after the addition of 40 mM n-Decyl-β-DMaltopyranoside (DM). Imidazole was then added to the lysate at 5 mM and bound for 1 h at RT with rotation, to 1 ml of Talon resin (Clontech), pre-equilibrated with 50 mM KPi pH 7.5, 150 mM NaCl, 5 mM Imidazole, and 40 mM DM. The protein-bound Talon was batch washed two times before loading onto a column and washed again with 10-column volumes of wash buffer containing 50 mM KPi pH 7.5, 150 mM NaCl, 40 mM imidazole, 4 mM DM. Protein was eluted in 6-column volumes with 50 mM KPi pH 7.5, 150 mM NaCl, 250 mM imidazole, 4 mM DM. Fractions were concentrated and buffer exchanged against imidazole with a 10,000 molecular weight cutoff concentrator (Amicon). Protein purity was determined by coomassie stain, and a protein assay was done to determine the concentration (Bio-Rad DC kit). Protein was used immediately or flash-frozen in liquid nitrogen and used within a week.

#### MTS-Peg5000 competition experiments

For all samples, 5 μg of protein in buffer containing 50 mM KPi pH 7.5, 150 mM NaCl, 4 mM DM, was added to 0.2 ml PCR tubes. For each sample, a group of conditions were set up in a volume of 20 μl (i) mock-buffer and protein only, (ii) Peg-only buffer and protein-only, (iii) experimental buffer and protein plus DHS at 250 μM, 375 μM, 500 μM, and 1mM, or Spectinomycin at 10 mM. Samples were vortexed, spun down, and incubated in a PCR block for 15 min at 22°C. For the mock sample, 1 μl of methanol only was added. Methoxypoly (ethyleneglycol)-5000-amidopropionyl-methanethiosulfonate (MTS-PEG5000) diluted in methanol and stored at −80°C was added to all remaining samples for a final concentration of 50 μM. Samples were then vortexed, spun down, and incubated again for 10 min in a 22°C PCR block. Reactions were stopped by adding 20 μl of nonreducing sample buffer with 10 mM Iodoacidamide for a final concentration of 5 mM. Samples were immediately loaded and ran on a 4%–20%, or any KD, Criterion 18-well gel. Standard western blot was performed with the primary antibody Penta.His (Qiagen) diluted to 1:4,000 in blocking buffer. The secondary antibody goat anti-mouse HRP (Bio-Rad) was used at 1:70,000 diluted in blocking buffer. Blots were developed with Immobilon HRP substrate (Millipore) and exposed to film. Experiments were repeated at least three times for each construct.

### Electrophysiology

Giant spheroplasts were generated from the *E*. *coli* strain PB104 (*ΔmscL*::*Cm*) [31] or MJF612 [56] and used in patch-clamp experiments as described previously [57]. Excised, inside-out patches were examined at RT under symmetrical conditions using a buffer comprised of 200 mM KCl, 90 mM MgCl_2_, 10 mM CaCl_2_, and 5 mM HEPES pH 6–7 (Sigma, St. Louis, MO). Recordings were performed at −20 mV (positive pipette). Data were acquired at a sampling rate of 20 kHz with a 5 kHz filter using an AxoPatch 200B amplifier in conjunction with Axoscope software (Axon Instruments, Union City, CA). A piezoelectric pressure transducer (World Precision Instruments, Sarasota, FL) was used to monitor the pressure throughout the experiments. As previously described [13,32,57], MscS was used as an internal standard for determining MscL sensitivity. Measurements were performed using Clampfit10 from Pclamp10 (Axon Instruments, Union City, CA).

### Computational Methods

#### Homology modeling

The all-atom structures of Ec-MscL were generated with MODELLER 9.13 [58] using the crystal structure of Mt-MscL (PDB Code 2OAR) as the template. Two sequence alignments, one was generated by PROMALS3D [59], and the other reported by Chang et al. [9], were applied in the homology modeling process. For each alignment, the best of 100 homology models based on the DOPE scores was selected for further evaluation. The selected homology models were assessed using the PROTABLE module of SYBYL software: (https://www.certara.com/products/molmod/sybyl-x).

The sequence alignment generated with PROMALS3D is shown in S11 Fig. The Ramachandran plots are also shown in S11 Fig. As the homology model based on the PROMALS3D alignment has fewer severe and minor violations, it was selected as the starting point of the further modeling work.

#### System setup

CHARMM-GUI (www.charm-gui.org) was applied to add POPC (1-palmitoyl-2-oleoyl-sn-glycero-3-phosphocholine) (S12 Fig) lipid bilayer, count ions, and water molecules. Note that MscL is neutral and DHS has a net charge of +3. We did not add counter ions to neutralize DHS in most of our simulations, as is sometimes performed in MD studies, since it is less physiological to simulate the passage of DHS through the Ec-MscL channel using our protocol, i.e., the electric field was applied only to DHS. The whole system consisted of one copy of Ec-MscL homopentamer, 230 POPC, 95 K^+^, 95 Cl^-^, and 32368 TIP3P [60] water molecules. In total, there were 138,947 atoms in the simulation box for ligand-free system. When DHS was introduced, the whole simulation system had 138,858 atoms.

As to force field parameters, the partial atomic charges of DHS were derived by RESP [61] to fit the HF/6-31G* electrostatic potentials generated using the GAUSSIAN 09 software package [62]. The other force field parameters came from GAFF in the AMBER12 [63]. The residue topology of DHS was prepared using the ANTECHAMBER module in AMBER 12 [64]. The AMBER FF12SB [65] and LIPID14 [66] force fields were used to model proteins and lipids, respectively.

#### Implementation of the function of molecular mechanical (MM) simulation with EEF

We modified the AMBER PMEMD code so that EEF can be applied to a subset of atoms during both minimizations and MD simulations. Atomic forces ($f_{i}^{X}$,$f_{i}^{Y}$,$f_{i}^{Z}$) due to the EEF are calculated with Eq 1, where *c*_*i*_ is the partial charge of atom *i*, EEF^X^, EEF^Y^, EEF^Z^, are the external electric field strength (Volt/Å) in the X, Y, and Z directions, respectively.

$$\begin{array}{l} f_{i}^{X}=c_{i}\times EEF^{X}\\ f_{i}^{Y}=c_{i}\times EEF^{Y}\\ f_{i}^{Z}=c_{i}\times EEF^{Z} \end{array}$$ (1)

#### MD simulations

All MD simulations were performed with periodic boundary condition to produce isothermal-isobaric ensembles using the modified PMEMD.CUDA program in AMBER 12 [63]. The Particle Mesh Ewald (PME) method [67–69] was used to calculate the full electrostatic energy of a unit cell in a macroscopic lattice of repeating images. All bonds were constrained using the SHAKE algorithm [70] in MD simulations. Temperature was regulated using the Langevin dynamics [71] with the collision frequency of 5 ps^-1^ [72–74]. Pressure was regulated using the isotropic position scaling algorithm with the pressure relaxation time set to 1.0 picosecond. The integration of the equations of motion was conducted at a time step of 1 femtosecond for the relaxation and equilibrium phases and 2 femtoseconds for the sampling phases.

Prior to MD simulations, the systems were relaxed to remove any possible steric crashes by a set of 10 thousand-step minimizations with the main chain atoms restrained. The harmonic restraint force constants decreased from 20 to 10, 5, and 1 kcal/mol/ Å^2^, progressively. At last, the systems were further relaxed by a 10,000-step minimization without any constraint or restraint.

There are three phases in a typical MD simulation, namely, the relaxation phase, the equilibrium phase, and the sampling phase. In the relaxation phase, the main chain atoms were gradually relaxed by applying a series of restraints, and the force constants decreased progressively: from 20 to 15, 10, 5, 2 and 1 kcal/mol/Å^2^. For each force constant, the position-restrained MD simulation was run for 1 nanosecond. In the following equilibrium phase, the system was further equilibrated for 6 nanoseconds without any restraint and constraint. In the sampling phase, snapshots were saved at an interval of 4 picoseconds for postanalysis. The postanalysis was conducted using the PTRAJ module of AMBER12 [63]. Special cases are presented below.

In order to study how DHS passes through the Ec-MscL channel, we first performed manual docking to get the starting points for MD simulation. For each docking orientation, a 50 nanoseconds, MD simulation was performed with secondary structure domains (TM1, TM2, S1, and C-terminal helix) and lipids restrained with a high harmonic force (force constant = 50 kcal/mol/Å^2^). Then, a 100 nanosecond MD simulation was carried out without any constraint or restraint.

In order to observe the DHS passing thought Ec-MscL event in a short time scale MD simulation, an EEF was applied to the DHS. Different EEF strengths, from 0.01 to 0.2 volt/Å were tested. With the EEF strength being set to 0.2 volt/Å along the Z-axis, we observed the complete procedure of the pass-through event within 10 nanoseconds (Fig 4B). However, we couldn’t observe the whole passing through procedure even at 300 nanoseconds when the EEF strength was reduced to 0.1 volt/Å.

The stability of MD trajectories was demonstrated with the plots of backbone RMSD along the simulation trajectories. As shown in S13 Fig, the MD trajectories of both ligand-free Ec-MscL and Ec-MscL/DHS are stable after a short period of equilibration. The residues participating least-squares fitting include two transmembrane domains (TM1 and TM2), the N-terminal cytoplasmic helix (S1), and the C-terminal cytoplasmic helix.

To exclude the possibility of DHS passing through the Ec-MscL channel by chance, we repeated the passing-through simulations five times using different random seeds to assign the initial velocities of atoms. We found that all the five simulations are extraordinarily consistent.

#### MM-PB energy calculation

For an Ec-MscL/DHS complex structure, the MM energy (E_MM_), and the polar part of the solvation-free energy was calculated without any minimization. All energy terms, including the bonded terms, were included in the MM calculations. The polar part of the solvation-free energy, E_PB_, was calculated by solving the finite-difference Possion-Boltzmann equation using AMBER12 [63]. The total MM-PB energy is the sum of E_MM_ and E_PB_.

As MscL is a membrane protein, two external dielectric constants, 80 for water and 4 for the membrane were used. The membrane thickness was simply set to 18Å as the average value of 4,000 snapshots was 18.1 ± 0.2 Å. Since the geometric center is not necessarily the center of the membrane, an offset along the Z-axis was applied to shift the center of the finite difference grids. This offset, called mctrdz in AMBER, was calculated for every individual conformation. The nonpolar part of the solvation energy, which is usually called the surface area (SA) term, was not calculated in this study due to the fact the available surface tension parameters were developed mainly for the nonmembrane systems.

#### Binding site identification

The possible binding sites of DHS to Ec-MscL was identified using the SiteID package implemented in Sybyl-X2.1.1. Only those having cavity volumes larger than 50 Å^3^ were kept. Three binding sites satisfy the above criterion for the last snapshot of the ligand-free MD simulation.

#### Docking protocols

Two types of docking were performed in this study. First of all, we manually placed DHS into the center of the open pore of Ec-MscL. Ten docking poses, which represent ten different orientations of DHS, were prepared for the following MD simulations without EEF. The conformation with the best energy was selected to study the passing-through event.

In the second type of docking, GLIDE of Schrodinger LLC [34] was applied to dock DHS to the three binding pockets identified by SiteID. The default parameters were used except for the scaling factor of *van der Waals* radii changed from 0.8 to 0.76 in order to slightly weaken the strong unfavorable repulsion between the ligand and receptor. Meaningful GLIDE scores were obtained only for the magenta binding site in Fig 3A, and the best GLIDE score is −7.46 kcal/mol. No meaningful docking score was resulted for the cyan and green docking poses.

#### Contact number calculation

To understand the role of N103 in the MscL gating, we measured the numbers of lipid hydrophobic residues that have close contact with N103 and its counterparts in other monomers, i.e., N239, N375, N511, and N647. Each POPC lipid consists of three residues, which are PA, PC, and OL (S12 Fig). PA and OL are hydrophobic residues. A PA or OL residue is recognized to contact with N103 or its counterparts only when the distance of the geometric centers of the lipid residue and N103 or its counterparts are equal to or smaller than a threshold. We have tried several thresholds, 5.5, 6.5, 7.5 and 8.5 Å. The results are similar and only those obtained with the threshold of 6.5 Å were reported.

#### Ec-MscL pore radius calculation

To find out how DHS open the MscL channel, we calculated the pore radii along the channel coordinate for the representative conformations at the five stages using the Hole2 software package (www.smartsci.uk). The C-terminal cytoplasmic helices were truncated for the sake of simplicity.

## Supporting Information

S1 Data Excel spreadsheet containing, in separate sheets, the underlying numerical data and statistical analysis for figure panels in Figs 2B and 4B–4D, and S2B, S2C, S3, S7, S9B, S11B–S11D and S13A–S13D Figs. The numerical data used in all figures and Tables 1 and 2 are included here.
(XLSX)

S1 Fig Spectinomycin fails to block the binding of MTS-PEG5000 to L19C and I25C at 10 mM. (A) Shown is a western blot analysis after a MTS-PEG5000 versus spectinomycin competition assay for the two mutations that showed positive results in the DHS competition assay. The absence (−) or presence (+) of 50 μM MTS-PEG5000 (MTS-PEG), as well as the absence (−) or presence of (+) 10 mM of spectinomycin, as indicated in the table at the bottom. There is no reduction in upper bands seen indicating no detectable inhibition of PEG modifications. (B) Shown are negative controls: WT Ec-MscL, which does not have any naturally occurring cysteine, shows no upper band with MTS-PEG5000 (left) and an unrelated Ec-MscL with a mutation in the periplasm, L47C (right) also shows no inhibition of the PEGylation by spectinomycin.
(TIF)

S2 Fig Wild type and mutated Ec- and Hi-MscL channels are functional both in vivo and as assayed by patch clamp. *(A)* The activity of different MscL channels was compared in patch clamp experiments using the giant spheroplast preparation from *E*. *coli* strain pB104, which is null for MscL but still contains the unrelated mechanosensitive channel MscS used as an internal control. Representative traces of Ec-MscL, L19M Ec-MscL, Hi-MscL and M19L Hi-MscL channel activities, are shown with the upper traces corresponding to the current and lower traces the pressure applied to the patch. The pressure threshold at which the first openings of MscS and MscL channel activities were recorded and are marked in the traces. *(B)* The ratio between the threshold pressure (see [31,32]) needed to gate MscL and MscS was used to compare Ec-MscL (Ec WT), L19M Ec-MscL (Ec L19M), wild type Hi-MscL (Hi WT), and M19L Hi-MscL (Hi M19L) tension sensitivity. No statistically significant differences were observed between each wild type and mutated channel for each species. (C) Expressed MscL constructs have the ability to rescue the MJF455 osmotic sensitive strain when compared to cells expressing empty vector (Empty) in an in vivo down shock assay. *E*. *coli* MscL L19M (Ec L19M) is shown to be a very slight partial LOF when compared to *E*. *coli* MscL wild type (Ec Wt). No difference is seen with Hi-MscL wild type (Hi Wt) or *H*. *influenza* MscL M19L mutant (Hi M19L) when compared to *E*. *coli* wild type. *n* = 5, *p < .*05*, ****p < .00005 versus Eco MscL wild type 2-tailed *t* test.
(TIF)

S3 Fig MST analysis of DHS-MscL binding. Recombinant protein was labeled with the Monolith NT Protein Labeling Kit RED (Cat#L001) according to the supplied protocol. Labeled MscL was kept constant at 50 nM, all samples tested were diluted in a 20 mM HEPES (pH 7.4) and 1 mM DDM. After a 10 min incubation at RT, the samples were loaded into Monolith standard-treated capillaries, and the thermophoresis was measured at 25°C after 30 min incubation by a Monolith NT.115 instrument (NanoTemper Technologies, München, Germany). Laser power was set to 20% using 30 s on-time. The LED power was set to 80%. The dissociation Kd values were fitted by using the NTAnalysis software (NanoTemper Technologies, München, Germany). Isoniazid was used as a negative control and showed no affinity. The affinities are for DHS and **A:** M19L Hi-MscL (50.8 ± 4.6 μM). **B**: wild type *E*. *coli* MscL (9.81 ± 0.13 mM); **C**: K55T Ec-MscL (601 ± 55.1μM); Note that for a given protein, depending on the binding mode properties such as pKa, hydrophobicity, solubility, etc., the fluorescence change can be negative or positive (see [29]), as seen here with positive changes for Ec-MscL but negative for the Hi-MscL.
(PDF)

S4 Fig Identification of “hot spots” which are critical for the Ec-MscL/DHS binding using the representative Conformations. DHS is represented by spheres, the “hot spots” shown as blue sticks have strong interactions with DHS. (A) Conformation I. The residue IDs of “hot spots” include monomer 1: D53, K55, Q56, F57, A58, Q65, D67; monomer 2: R198(62), G202(66), D203(67), I204(68), P205(69), A206(70); monomer 3: D339(67); monomer 4: D471(63), A472(64); monomer 5: D611(67), I612(68), P613(69). (B) Conformation II. The residue IDs of “hot spots” include monomer 1: N81; monomer 2: V208(72); monomer 3: D339(67); monomer 5: A571(27), G574(30), K575(31), S578(34). (C) Conformation III. The residue IDs of “hot spots” include monomer 1: V23, G26, A27; monomer 2: V159(23), I160(24), A163(27); monomer 3: V295(23), I296(24), A299(27), A300(28), K303(31); monomer 4: V431(23); monomer 5: V567(23), I568(24), A571(27). Note that the residue IDs in parentheses are the corresponding residue IDs in Ec-MscL monomer.
(TIF)

S5 Fig A focused look at DHS and its surrounding residues in five stages. The hydrogen bonds between DHS and the surrounding residues are shown in magenta dashed-line. (A) Stage I, (B) Stage II, (C) Stage III, (D) Stage IV, (E) Stage V.
(TIF)

S6 Fig Comparison of DHS conformations in the five stages. (A) Stage I, (B) Stage II, (C) Stage III, (D) Stage IV, (E) Stage V.
(TIF)

S7 Fig The pore radii of MscL along the channel Z-coordinates. Shown are pore radii for the representative conformations of five stages (Left panel). The right panel is the cartoon representation of the representative conformation of Stage IV with the channel path represented by green dots. The magenta spheres indicate the channel Z-coordinates from −60 to 0 at a step of 10 Å. The 0 coordinate is the center of five K106 LYS residues in blue sticks (LYS 106, 242, 378, 514, and 650).
(TIF)

S8 Fig Competition experiments at additional residues predicted by MD snapshots to interact with DHS. *(A)* Western blot analysis after a competition experiment on the purified protein for residues predicted to interact with DHS, using the cysteine mutations indicated. The absence (−) or presence (+) of 50 μM MTS-PEG5000 (MTS-PEG), as well as the absence (−) or concentration of DHS used, is indicated in the table at the bottom. There is a reduction in upper bands seen in the two higher concentrations of DHS (500 μM and 1 mM), indicating PEG modifications. Note that the upper band of the doublet seen in A114C, T116C, and K117C, which appears to be due to dimer formation of the protein and varies according to proximity of the residues within the complex, can be seen in all samples including mock lanes containing no MTS-PEG5000. *(B)* Western blot analysis after a competition experiment with the purified protein of K106C and A110C, which are predicted to be negative. Upper bands, above the PEG-modified MscL, can be seen for all concentrations of DHS (250 μm–1 mM), indicating a slight dimerization. *(C)* N103C, predicted not to interact with DHS, gives a positive result in this assay. However, as discussed in text, this residue (103) has been shown to become buried within the hydrophobic lipids upon channel gating; thus, this positive result may actually be from an inaccessibility due to a conformational change leading to its burial in a hydrophobic environment, not because it is involved in the binding site.
(TIF)

S9 Fig DHS increases Ec- MscL channel activity as assayed by patch clamp in native membranes. The activity of Ec-MscL channels was studied in patch clamp experiments using giant spheroplast preparations from the *E*. *coli* strain MJF 612, which is null for four mayor bacterial MS channels. (A) Representative traces of a single Ec-MscL patch before (left panel) and after treatment with 100 μM DHS (right panel). For these experiments, a backfilled pipette technique was used, where the tip contains buffer without compound and the rest of the electrode contains buffer with compound. This technique allows the patch to see the drug from the extracellular side only after diffusion of the compound from the electrode to the tip (which normally takes 10 to 20 min). Upper traces show the current with upward deflections reflecting the channel openings; the negative pressures at which the patch was held is shown in the lower portion of the traces. Before and after traces belong to the same patch after incubation for a minimum of 30 min to allow for the diffusion of DHS to the pipette tip. (B) Graphs comparing the NP_o_ of Ec-MscL channels at time zero and after at least 30 min in control conditions (no DHS, left panel) or with 100 μM DHS in the pipette (right panel).
(TIF)

S10 Fig Comparison of the binding site conformations in the five stages. Alignments were made using only main chain atoms with the last snapshot of ligand-free MD simulation as the reference structure (in magenta sticks). (A) Stage I, (B) Stage II, (C) Stage III, (D) Stage IV, (E) Stage V.
(TIF)

S11 Fig Homology modeling of Ec-MscL. (A) Sequence alignment generated using PROMALS3D. (B), (C), and (E) are the Ramachandran plots of MscL models: the magenta dots indicate minor violations (PRO in allowed region and non-GLY residues in generously allowed region), and red suggest severe violations (PRO in generously allowed region and non-GLY residues in disallowed region) for the Phi-Psi torsional angles. (B) Homology model based on the alignment generated by PROMALS3D: 13 red and 17 magenta dots. (C) Homology model based on the alignment by Chang et al. (Science, 282, 2220–2226, 1998,): 18 red and 29 magenta dots; (D) Crystal structure of Mt-MscL (PDB Code 2OAR, resolution 3.5 Å): 22 red and 16 magenta dots. (E) The residues with severe violations represented by magenta spheres are mainly located on the loop areas for the PROMAL3D homology model.
(TIF)

S12 Fig Chemical structure of POPC lipid. POPC consists of three residues which are PA (blue), OL (red) and PC (black).
(TIF)

S13 Fig RMSD of α-carbon of amino acids along the time course of MD simulations. (A) ligand-free MD simulation, the reference structure for RMSD calculation is the crystal structure of Mt-MscL (PDB Code 2OAR). (B) MD simulation of DHS passing through the MscL channel without EEF applied. Red curve represents the RMSDs with the crystal structure of Mt-MscL as the reference, while black curve represents the RMSDs with the last snapshot of the ligand-free MD simulation as the reference. (C) MD simulation of DHS passing through the MscL channel with an EEF strength of 0.2 volt/Å applied to the DHS. Least-squares fitting with the last snapshot of the ligand-free MD simulation of Ec-MscL as the reference structure was first performed for all the α-carbons of Ec-MscL, and then the RMSDs of secondary structure domains (TM1, TM2, TM1+TM2, S1, and C-terminal helix) were calculated directly without fitting. (D) Similar to (c) except that the reference structure in least-squares fitting is the last snapshot of Ec-MscL/DHS MD simulation without EEF being applied.
(TIF)

S1 Table List of ΔZ and RMSDs for four typical conformations sampled during the passing-through event. ΔZ is the distance between the ligand and five LYS residues (K106, K242, K378, K514, and K650) along the Z-axis; RMSDs are root-mean-squared deviations of α-carbon for all and major secondary-structure domains.
(XLSX)

S2 Table The axis angle representations of the rotation matrices generated by least-square fitting of the S1 (A), TM1 (B), TM2 (C), and C-terminal (D) helices. The reference structure of least-square fitting is a closed conformation (the last snapshot of the ligand-free MD simulation).
(XLSX)

S1 Video A MD simulation video showing the DHS passing through the MscL pore, as described in text and reflecting the dynamic data from which the data in Fig 4 was derived. (MPG)

## Abbreviations

CphM: citrate-phosphate-defined media
DHS: dihydrostreptomycin
DM: n-Decyl-β-DMaltopyranoside
Ec-MscL: *Escherichia coli* MscL
EEF: external electric field
E_MM_: molecular mechanical energy
GLIDE: Grid-based Ligand Docking with Energetics
GOF: gain of function
Hi-MscL: *Haemophilus influenza* MscL
LB: Lennox broth
LOF: loss of function
MD: molecular dynamics
MM: molecular mechanical
MST: MicroScale Thermophoresis
Mt-MscL: *Mycobacterium tuberculosis* MscL
MTS: methanosulfonate
PME: Particle Mesh Ewald
RMSD: root-mean-square deviation
RT: room temperature
SA: surface area
TM1: first transmembrane domain
TM2: second transmembrane domain
