# Supplementary figures and images for: Dihydrostreptomycin Directly Binds to, Modulates, and Passes through the MscL Channel Pore
Source: PLoS Biol. 2016 Jun 9;14(6):e1002473. doi: 10.1371/journal.pbio.1002473 (PMC4900634; doi:10.1371/journal.pbio.1002473)

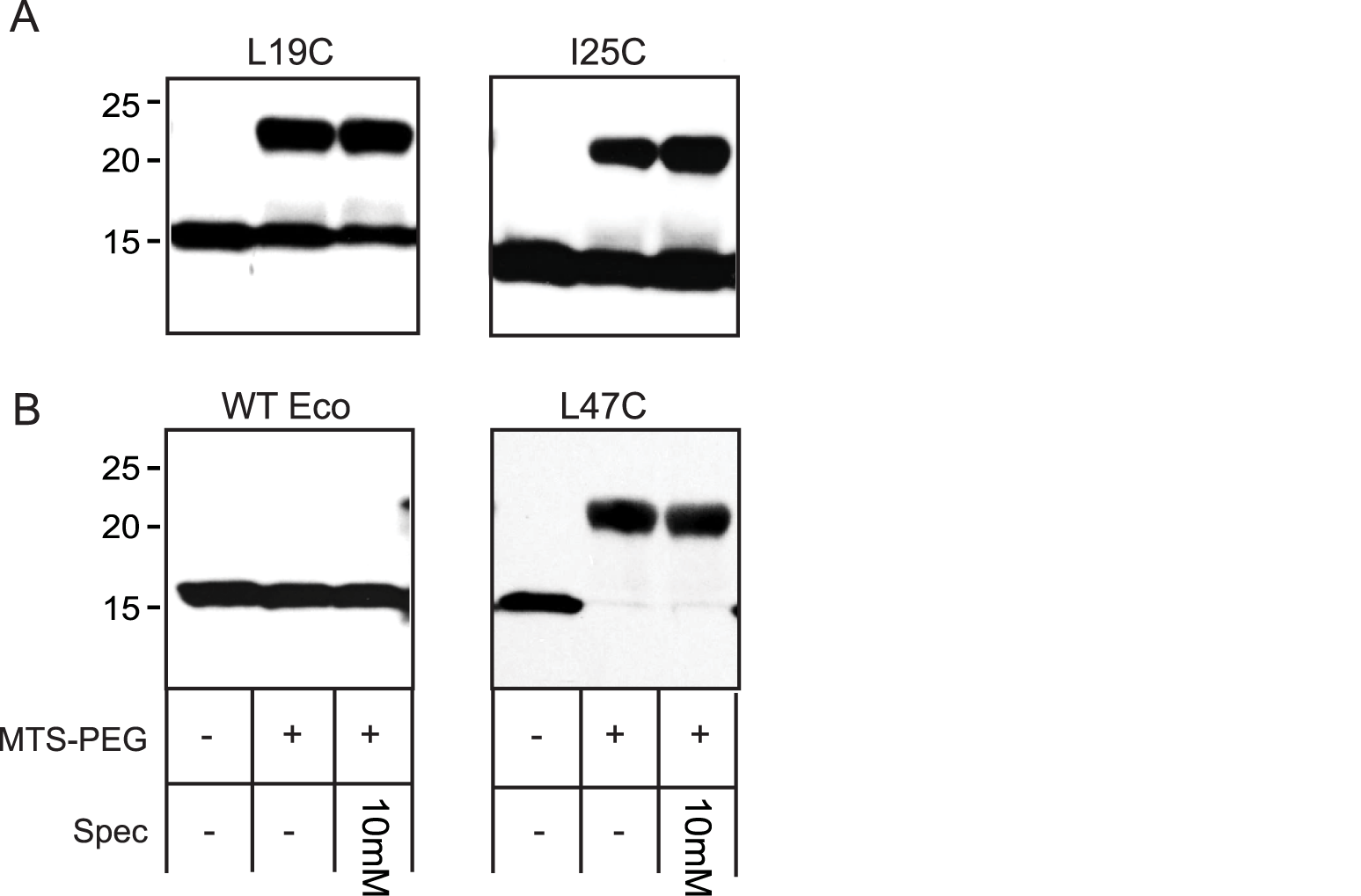

Supplement: S1 Fig — (A) Shown is a western blot analysis after a MTS-PEG5000 versus spectinomycin competition assay for the two mutations that showed positive results in the DHS competition assay. The absence (−) or presence (+) of 50 μM MTS-PEG5000 (MTS-PEG), as well as the absence (−) or presence of (+) 10 mM of spectinomycin, as indicated in the table at the bottom. There is no reduction in upper bands seen indicating no detectable inhibition of PEG modifications. (B) Shown are negative controls: WT Ec-MscL, which does not have any naturally occurring cysteine, shows no upper band with MTS-PEG5000 (left) and an unrelated Ec-MscL with a mutation in the periplasm, L47C (right) also shows no inhibition of the PEGylation by spectinomycin. (TIF) [file pbio.1002473.s002.tif]

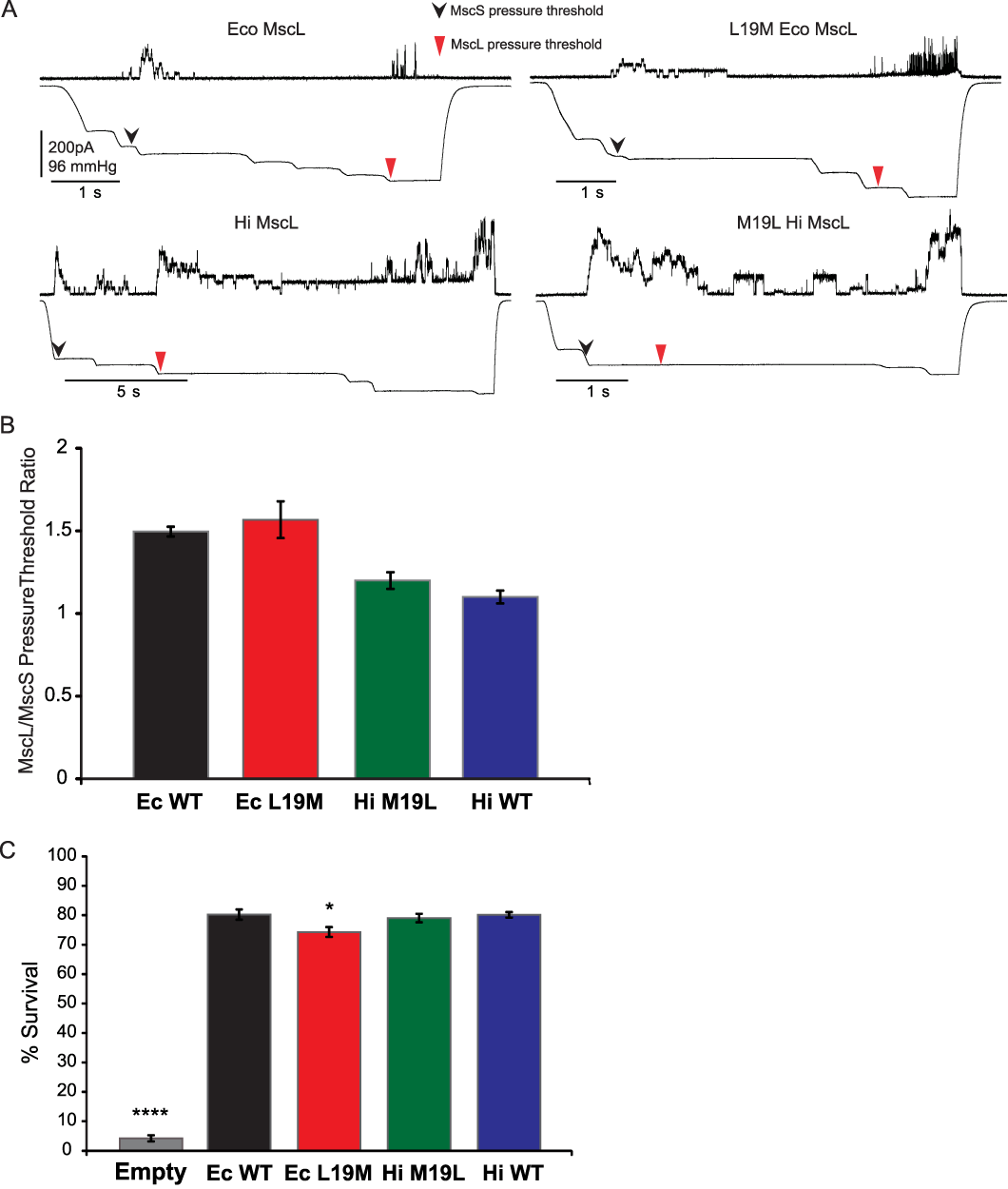

Supplement: S2 Fig — (A) The activity of different MscL channels was compared in patch clamp experiments using the giant spheroplast preparation from E. coli strain pB104, which is null for MscL but still contains the unrelated mechanosensitive channel MscS used as an internal control. Representative traces of Ec-MscL, L19M Ec-MscL, Hi-MscL and M19L Hi-MscL channel activities, are shown with the upper traces corresponding to the current and lower traces the pressure applied to the patch. The pressure threshold at which the first openings of MscS and MscL channel activities were recorded and are marked in the traces. (B) The ratio between the threshold pressure (see [31,32]) needed to gate MscL and MscS was used to compare Ec-MscL (Ec WT), L19M Ec-MscL (Ec L19M), wild type Hi-MscL (Hi WT), and M19L Hi-MscL (Hi M19L) tension sensitivity. No statistically significant differences were observed between each wild type and mutated channel for each species. (C) Expressed MscL constructs have the ability to rescue the MJF455 osmotic sensitive strain when compared to cells expressing empty vector (Empty) in an in vivo down shock assay. E. coli MscL L19M (Ec L19M) is shown to be a very slight partial LOF when compared to E. coli MscL wild type (Ec Wt). No difference is seen with Hi-MscL wild type (Hi Wt) or H. influenza MscL M19L mutant (Hi M19L) when compared to E. coli wild type. n = 5, *p < .05, ****p < .00005 versus Eco MscL wild type 2-tailed t test. (TIF) [file pbio.1002473.s003.tif]

A

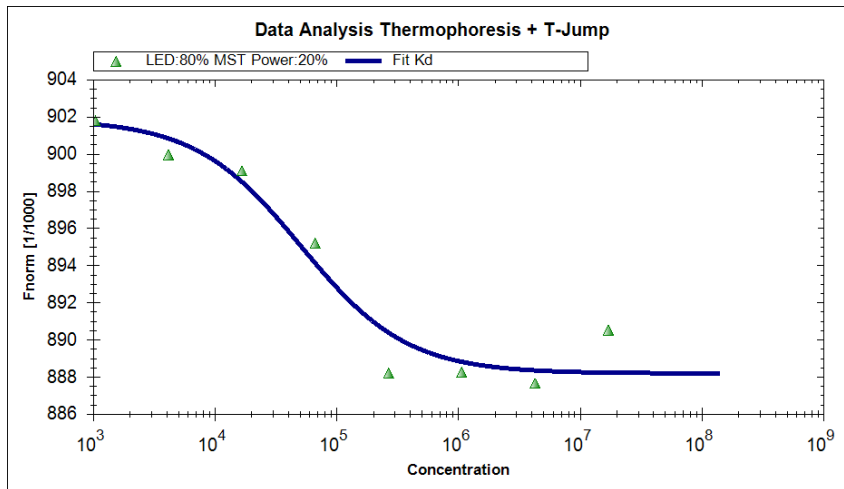

B

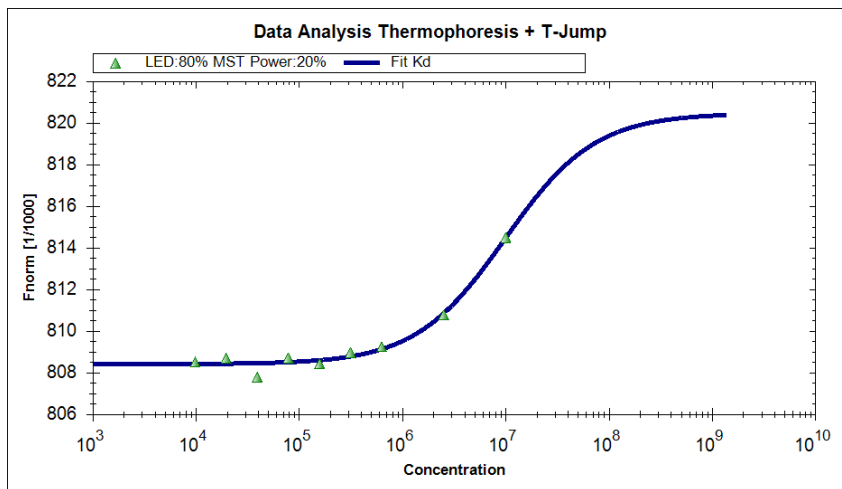

C

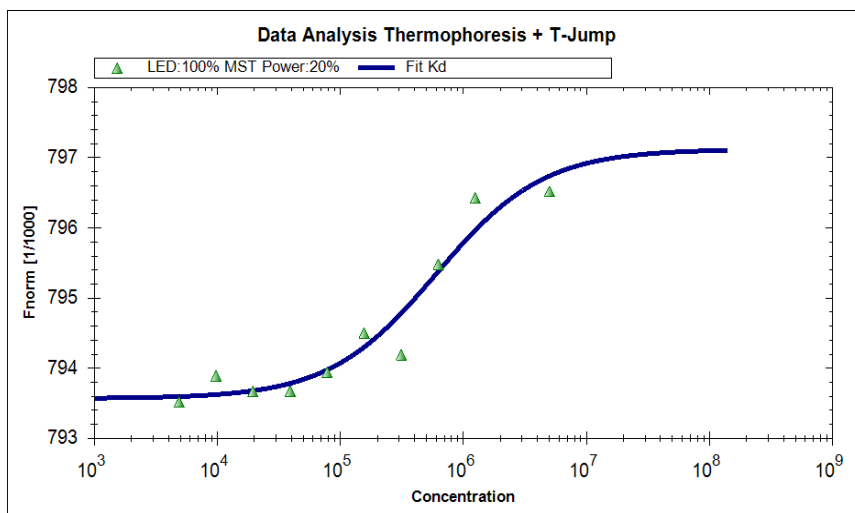

Supplement: S3 Fig — Recombinant protein was labeled with the Monolith NT Protein Labeling Kit RED (Cat#L001) according to the supplied protocol. Labeled MscL was kept constant at 50 nM, all samples tested were diluted in a 20 mM HEPES (pH 7.4) and 1 mM DDM. After a 10 min incubation at RT, the samples were loaded into Monolith standard-treated capillaries, and the thermophoresis was measured at 25°C after 30 min incubation by a Monolith NT.115 instrument (NanoTemper Technologies, München, Germany). Laser power was set to 20% using 30 s on-time. The LED power was set to 80%. The dissociation Kd values were fitted by using the NTAnalysis software (NanoTemper Technologies, München, Germany). Isoniazid was used as a negative control and showed no affinity. The affinities are for DHS and A: M19L Hi-MscL (50.8 ± 4.6 μM). B: wild type E. coli MscL (9.81 ± 0.13 mM); C: K55T Ec-MscL (601 ± 55.1μM); Note that for a given protein, depending on the binding mode properties such as pKa, hydrophobicity, solubility, etc., the fluorescence change can be negative or positive (see [29]), as seen here with positive changes for Ec-MscL but negative for the Hi-MscL. (PDF) [file pbio.1002473.s004.pdf]

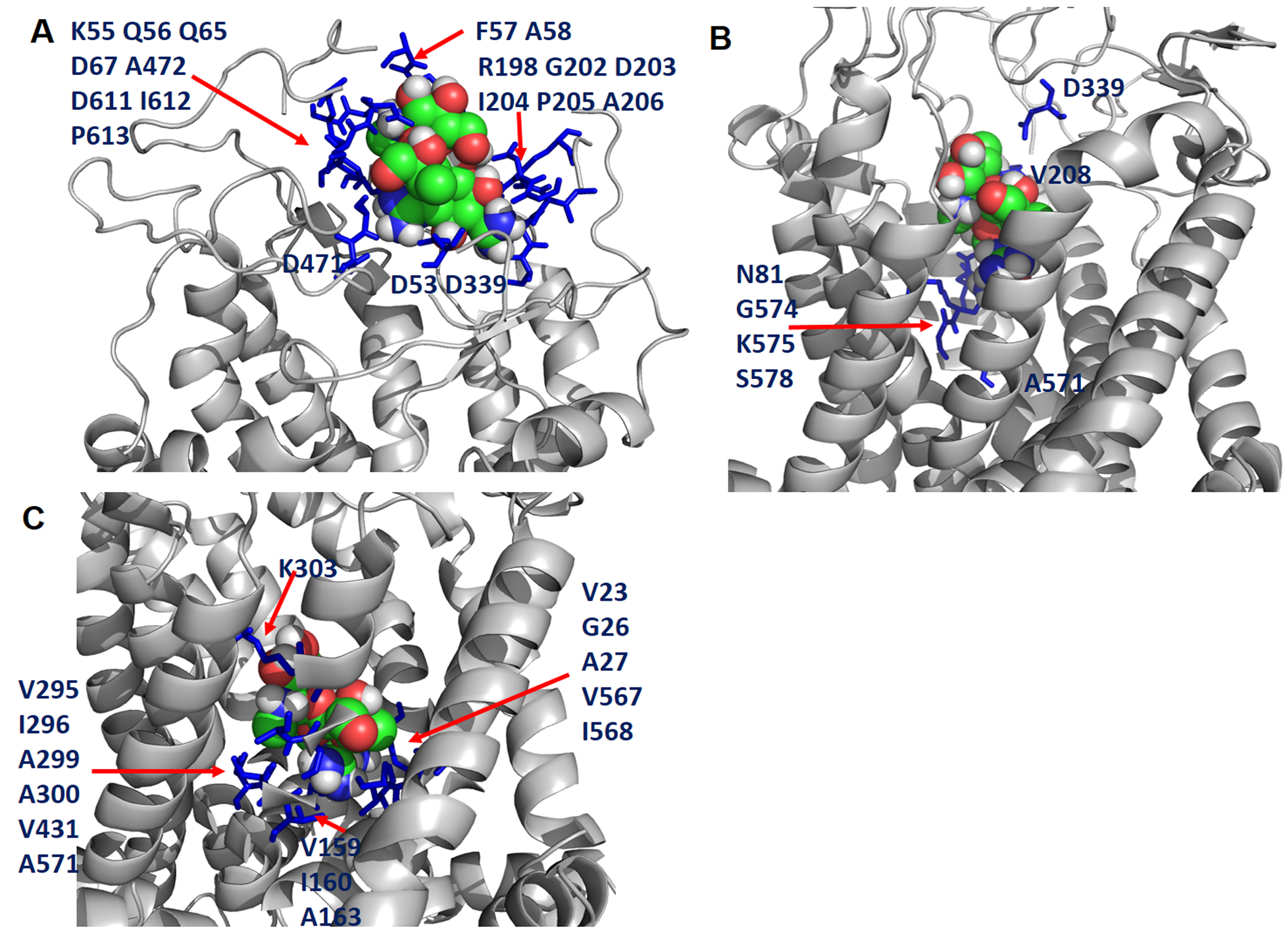

Supplement: S4 Fig — DHS is represented by spheres, the “hot spots” shown as blue sticks have strong interactions with DHS. (A) Conformation I. The residue IDs of “hot spots” include monomer 1: D53, K55, Q56, F57, A58, Q65, D67; monomer 2: R198(62), G202(66), D203(67), I204(68), P205(69), A206(70); monomer 3: D339(67); monomer 4: D471(63), A472(64); monomer 5: D611(67), I612(68), P613(69). (B) Conformation II. The residue IDs of “hot spots” include monomer 1: N81; monomer 2: V208(72); monomer 3: D339(67); monomer 5: A571(27), G574(30), K575(31), S578(34). (C) Conformation III. The residue IDs of “hot spots” include monomer 1: V23, G26, A27; monomer 2: V159(23), I160(24), A163(27); monomer 3: V295(23), I296(24), A299(27), A300(28), K303(31); monomer 4: V431(23); monomer 5: V567(23), I568(24), A571(27). Note that the residue IDs in parentheses are the corresponding residue IDs in Ec-MscL monomer. (TIF) [file pbio.1002473.s005.tif]

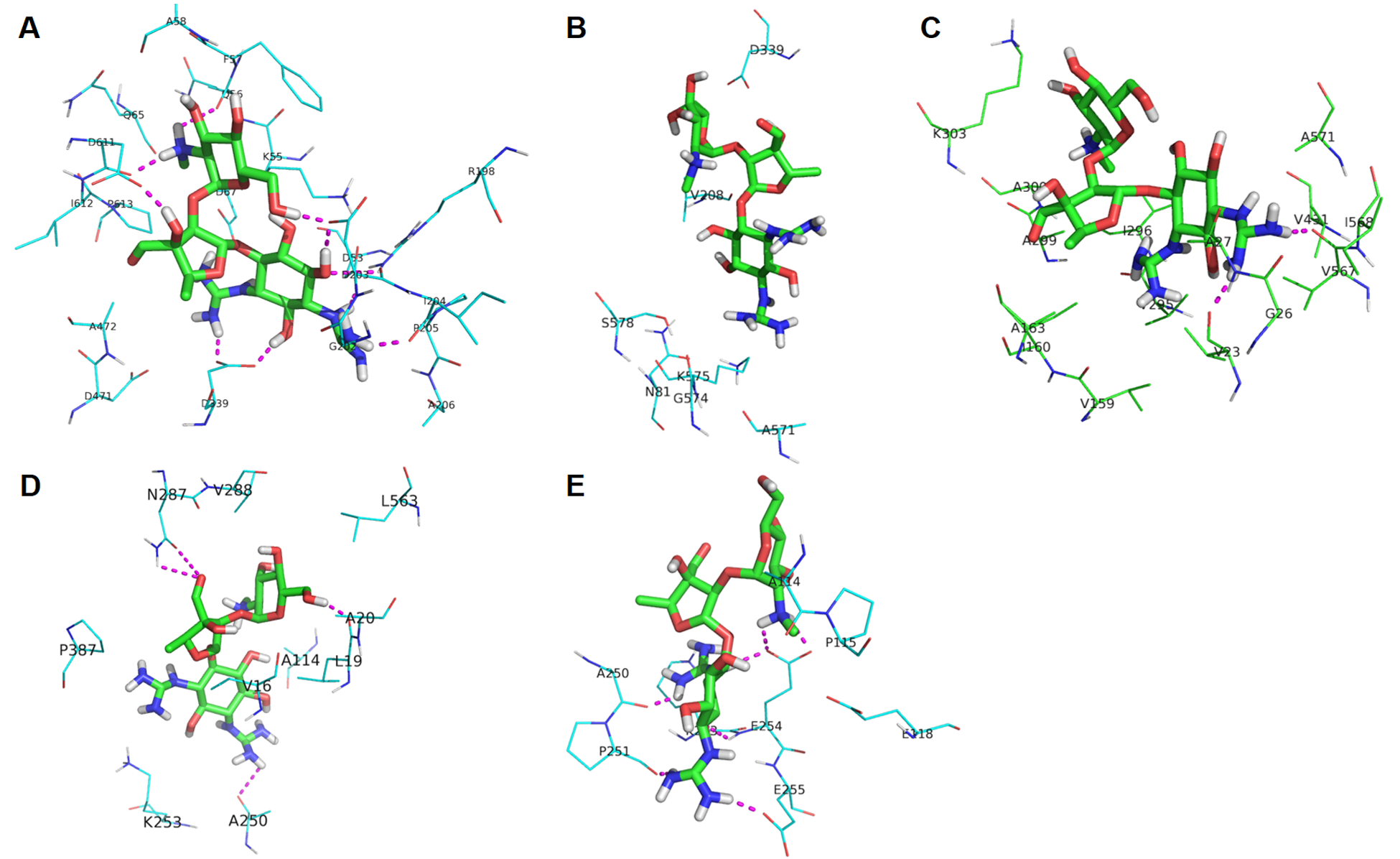

Supplement: S5 Fig — The hydrogen bonds between DHS and the surrounding residues are shown in magenta dashed-line. (A) Stage I, (B) Stage II, (C) Stage III, (D) Stage IV, (E) Stage V. (TIF) [file pbio.1002473.s006.tif]

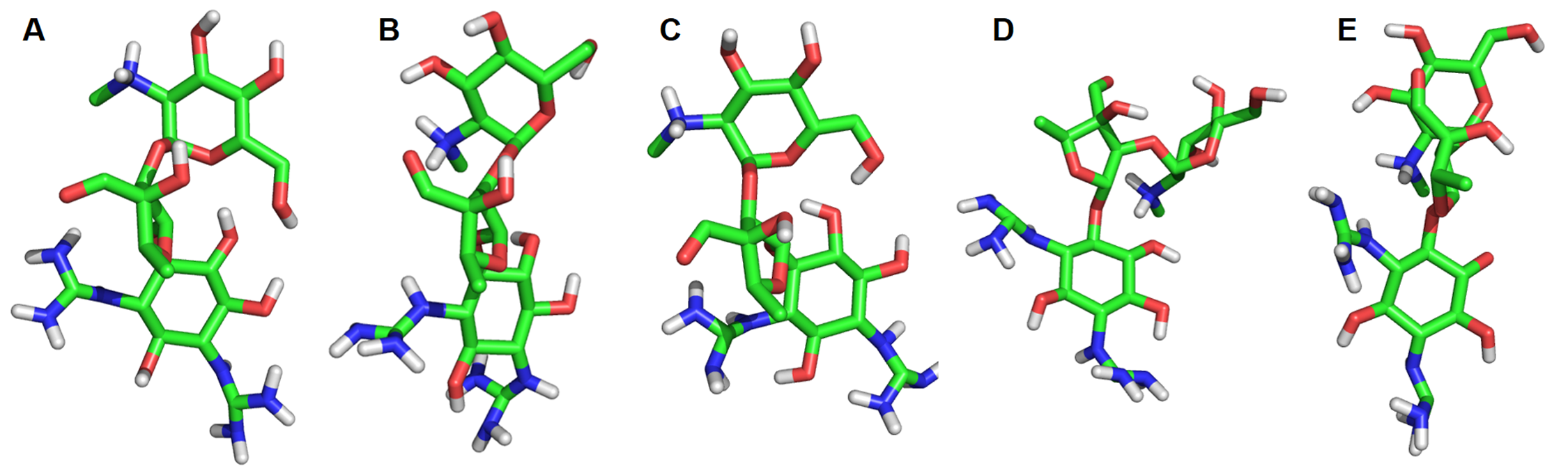

Supplement: S6 Fig — (A) Stage I, (B) Stage II, (C) Stage III, (D) Stage IV, (E) Stage V. (TIF) [file pbio.1002473.s007.tif]

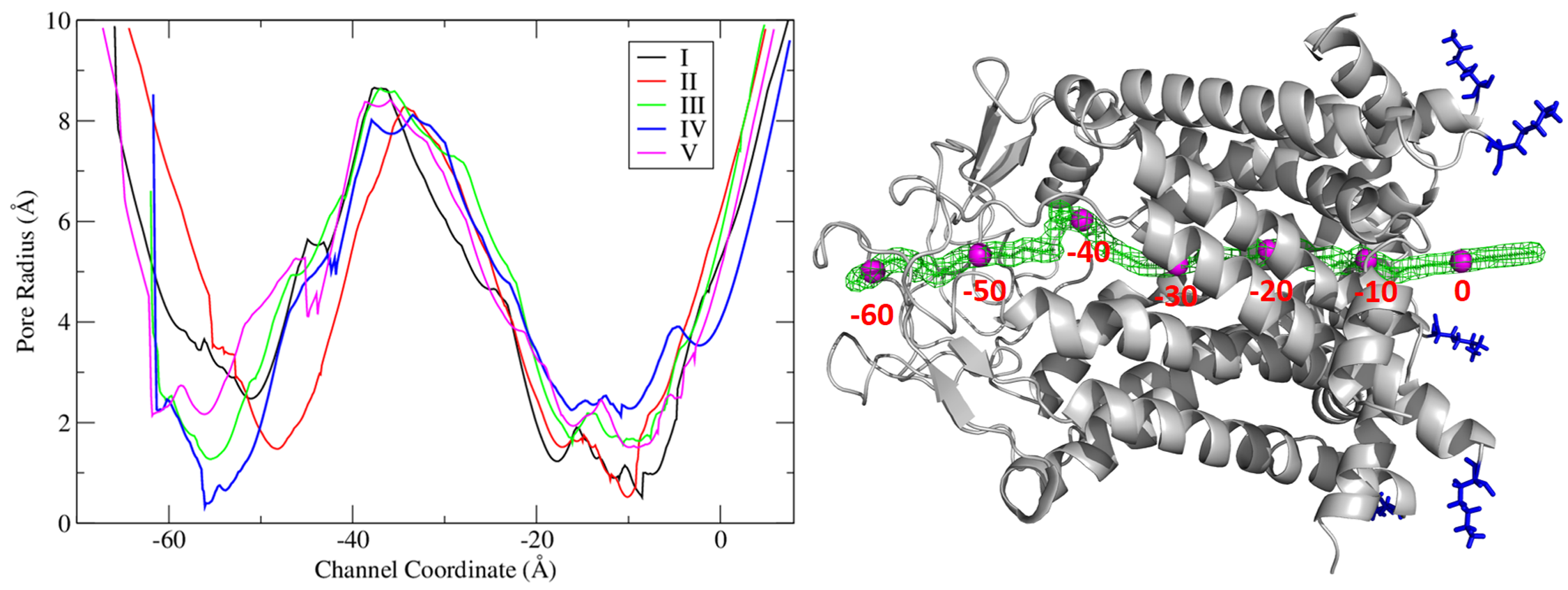

Supplement: S7 Fig — Shown are pore radii for the representative conformations of five stages (Left panel). The right panel is the cartoon representation of the representative conformation of Stage IV with the channel path represented by green dots. The magenta spheres indicate the channel Z-coordinates from −60 to 0 at a step of 10 Å. The 0 coordinate is the center of five K106 LYS residues in blue sticks (LYS 106, 242, 378, 514, and 650). (TIF) [file pbio.1002473.s008.tif]

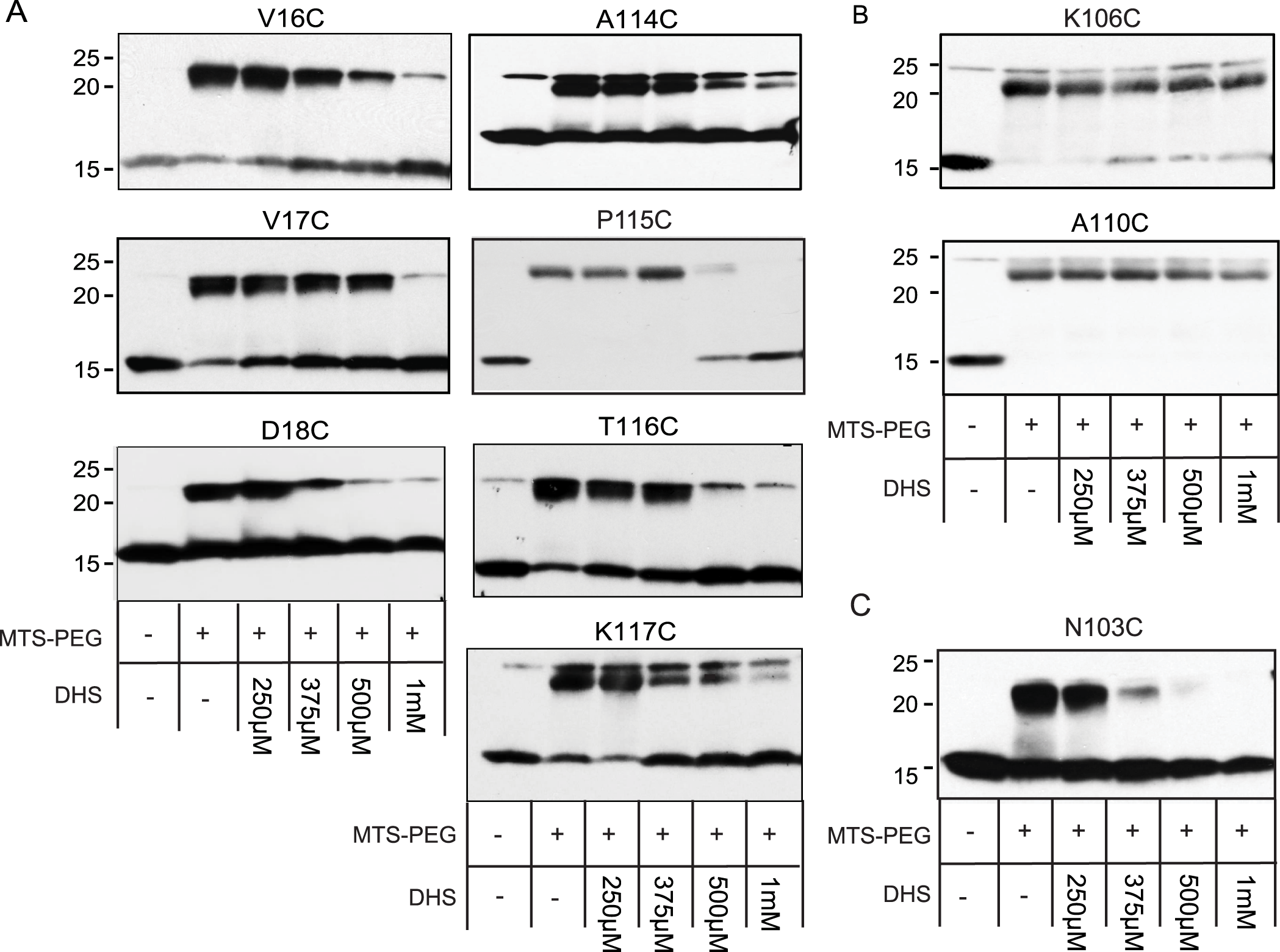

Supplement: S8 Fig — (A) Western blot analysis after a competition experiment on the purified protein for residues predicted to interact with DHS, using the cysteine mutations indicated. The absence (−) or presence (+) of 50 μM MTS-PEG5000 (MTS-PEG), as well as the absence (−) or concentration of DHS used, is indicated in the table at the bottom. There is a reduction in upper bands seen in the two higher concentrations of DHS (500 μM and 1 mM), indicating PEG modifications. Note that the upper band of the doublet seen in A114C, T116C, and K117C, which appears to be due to dimer formation of the protein and varies according to proximity of the residues within the complex, can be seen in all samples including mock lanes containing no MTS-PEG5000. (B) Western blot analysis after a competition experiment with the purified protein of K106C and A110C, which are predicted to be negative. Upper bands, above the PEG-modified MscL, can be seen for all concentrations of DHS (250 μm–1 mM), indicating a slight dimerization. (C) N103C, predicted not to interact with DHS, gives a positive result in this assay. However, as discussed in text, this residue (103) has been shown to become buried within the hydrophobic lipids upon channel gating; thus, this positive result may actually be from an inaccessibility due to a conformational change leading to its burial in a hydrophobic environment, not because it is involved in the binding site. (TIF) [file pbio.1002473.s009.tif]

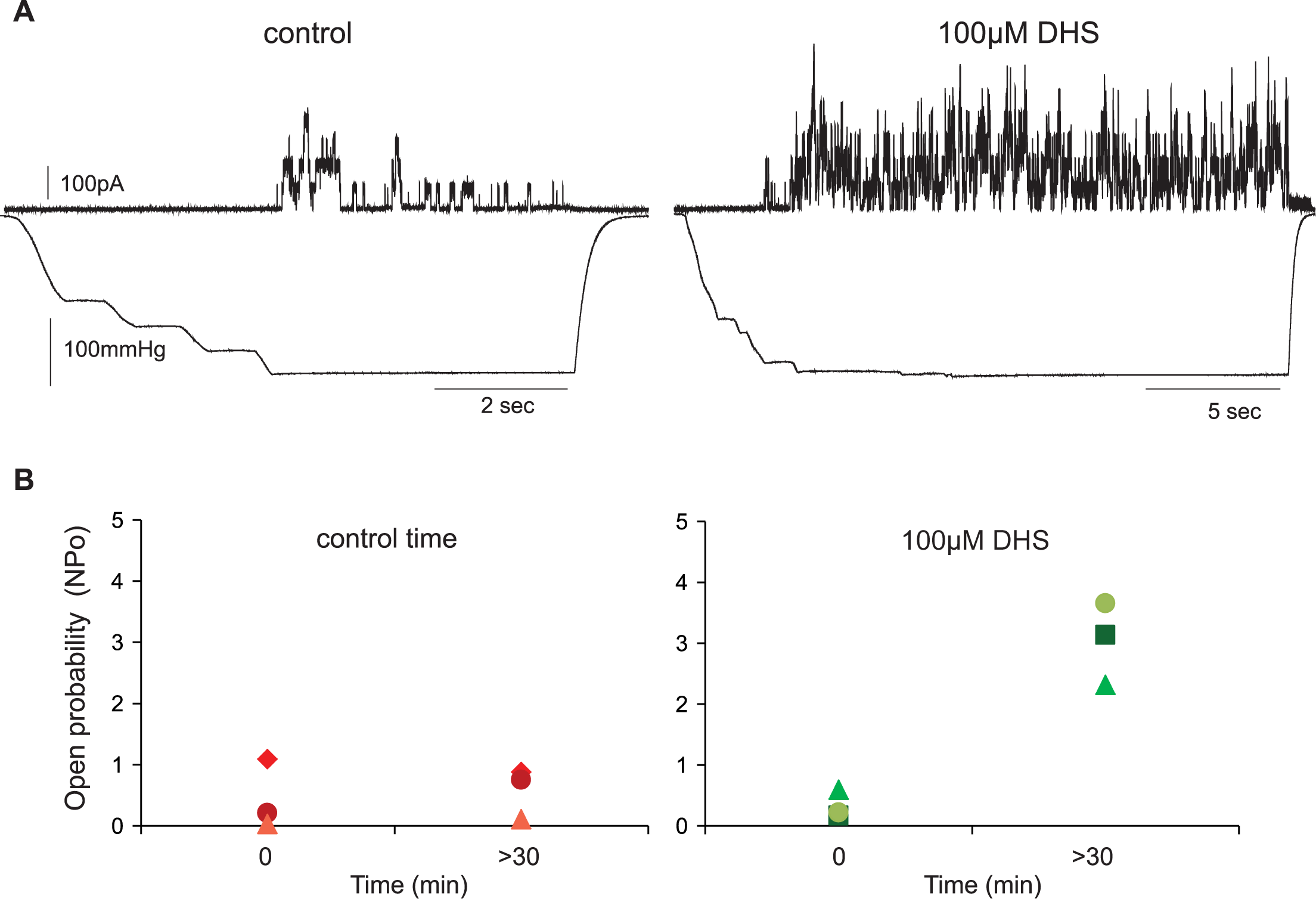

Supplement: S9 Fig — The activity of Ec-MscL channels was studied in patch clamp experiments using giant spheroplast preparations from the E. coli strain MJF 612, which is null for four mayor bacterial MS channels. (A) Representative traces of a single Ec-MscL patch before (left panel) and after treatment with 100 μM DHS (right panel). For these experiments, a backfilled pipette technique was used, where the tip contains buffer without compound and the rest of the electrode contains buffer with compound. This technique allows the patch to see the drug from the extracellular side only after diffusion of the compound from the electrode to the tip (which normally takes 10 to 20 min). Upper traces show the current with upward deflections reflecting the channel openings; the negative pressures at which the patch was held is shown in the lower portion of the traces. Before and after traces belong to the same patch after incubation for a minimum of 30 min to allow for the diffusion of DHS to the pipette tip. (B) Graphs comparing the NPo of Ec-MscL channels at time zero and after at least 30 min in control conditions (no DHS, left panel) or with 100 μM DHS in the pipette (right panel). (TIF) [file pbio.1002473.s010.tif]

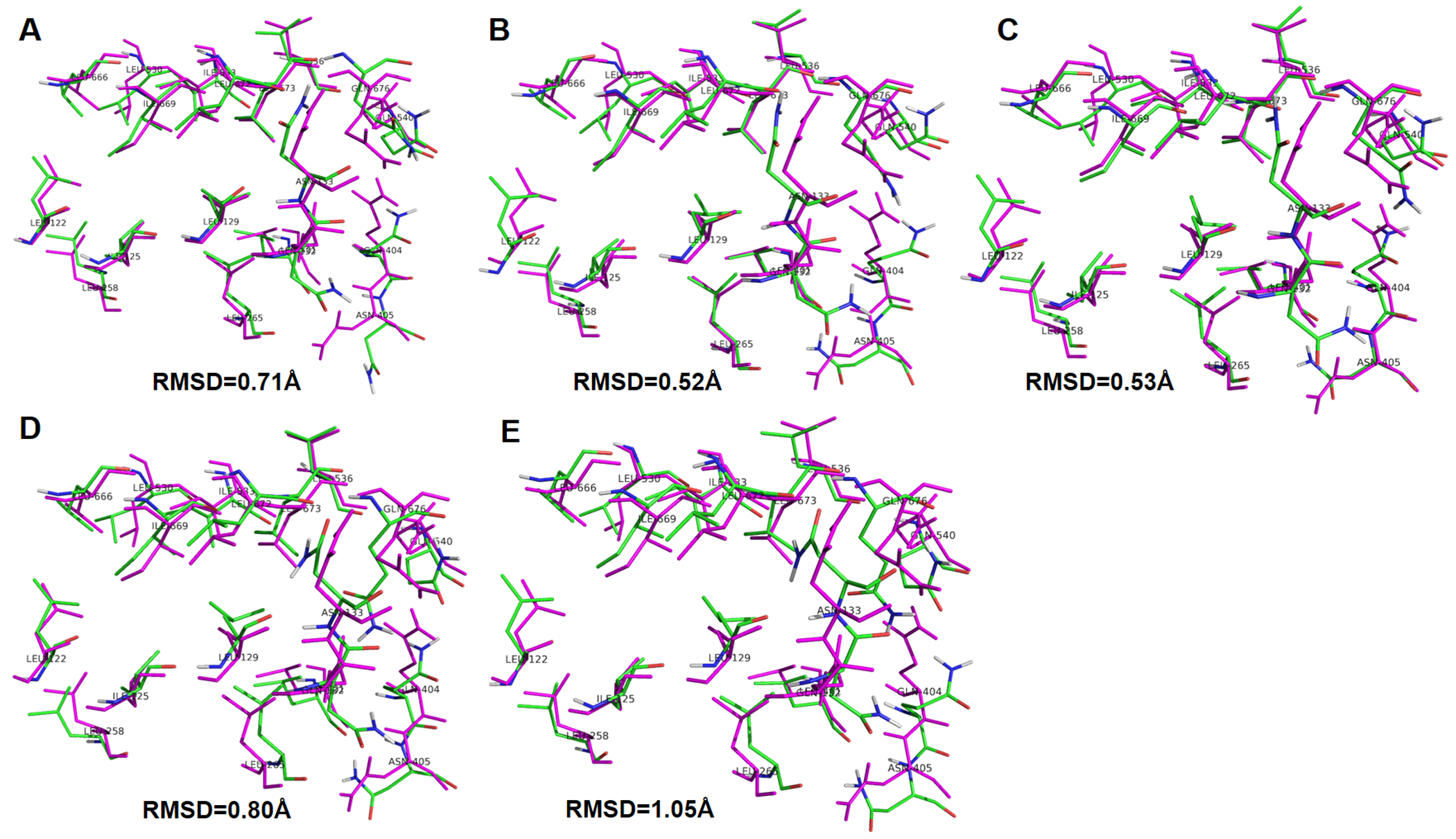

Supplement: S10 Fig — Alignments were made using only main chain atoms with the last snapshot of ligand-free MD simulation as the reference structure (in magenta sticks). (A) Stage I, (B) Stage II, (C) Stage III, (D) Stage IV, (E) Stage V. (TIF) [file pbio.1002473.s011.tif]

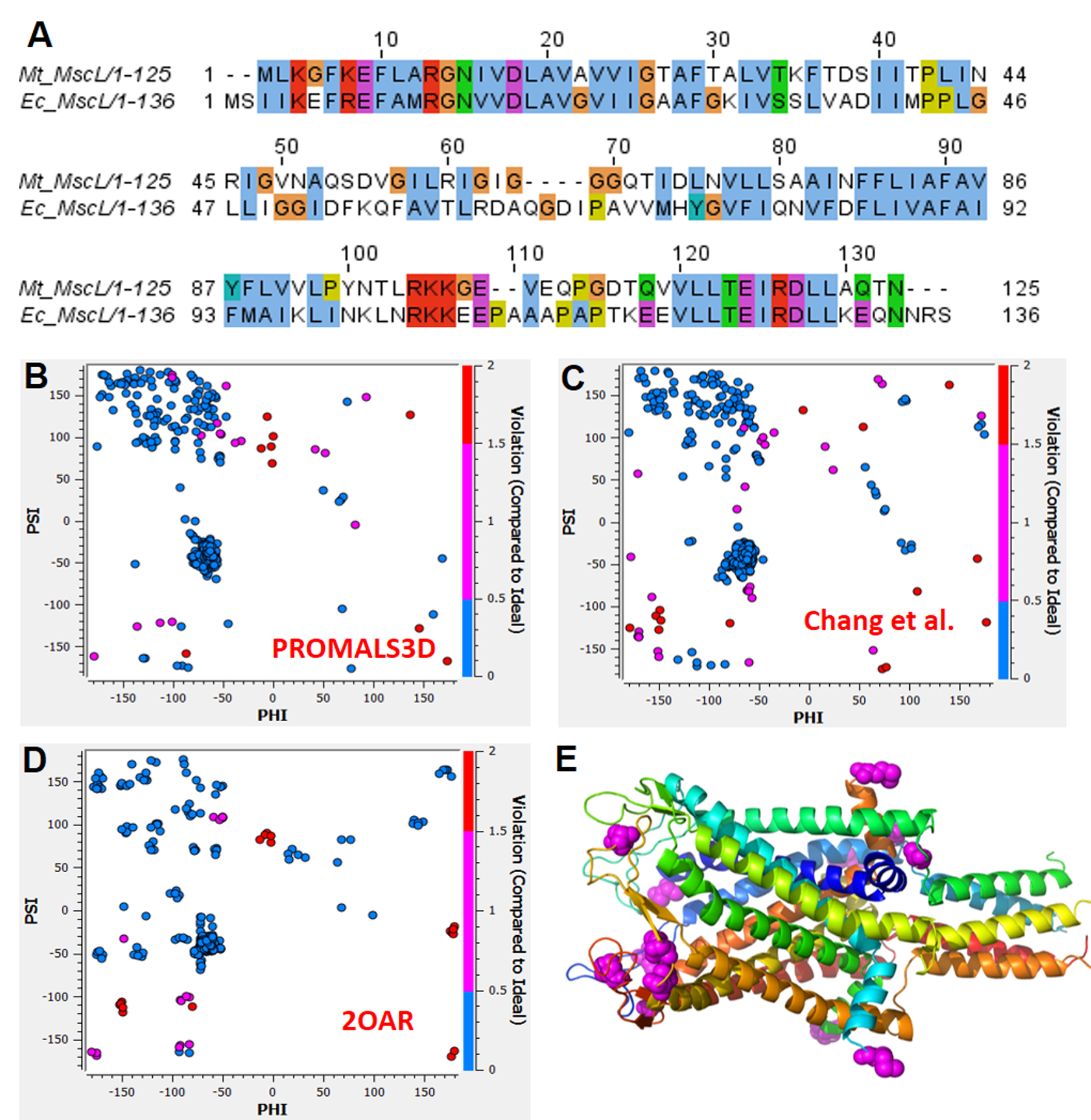

Supplement: S11 Fig — (A) Sequence alignment generated using PROMALS3D. (B), (C), and (E) are the Ramachandran plots of MscL models: the magenta dots indicate minor violations (PRO in allowed region and non-GLY residues in generously allowed region), and red suggest severe violations (PRO in generously allowed region and non-GLY residues in disallowed region) for the Phi-Psi torsional angles. (B) Homology model based on the alignment generated by PROMALS3D: 13 red and 17 magenta dots. (C) Homology model based on the alignment by Chang et al. (Science, 282, 2220–2226, 1998,): 18 red and 29 magenta dots; (D) Crystal structure of Mt-MscL (PDB Code 2OAR, resolution 3.5 Å): 22 red and 16 magenta dots. (E) The residues with severe violations represented by magenta spheres are mainly located on the loop areas for the PROMAL3D homology model. (TIF) [file pbio.1002473.s012.tif]

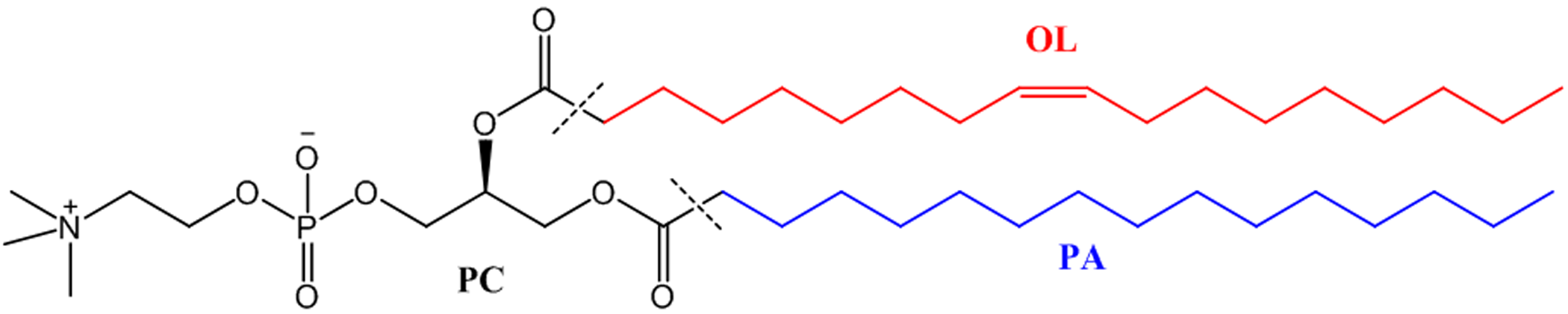

Supplement: S12 Fig — POPC consists of three residues which are PA (blue), OL (red) and PC (black). (TIF) [file pbio.1002473.s013.tif]

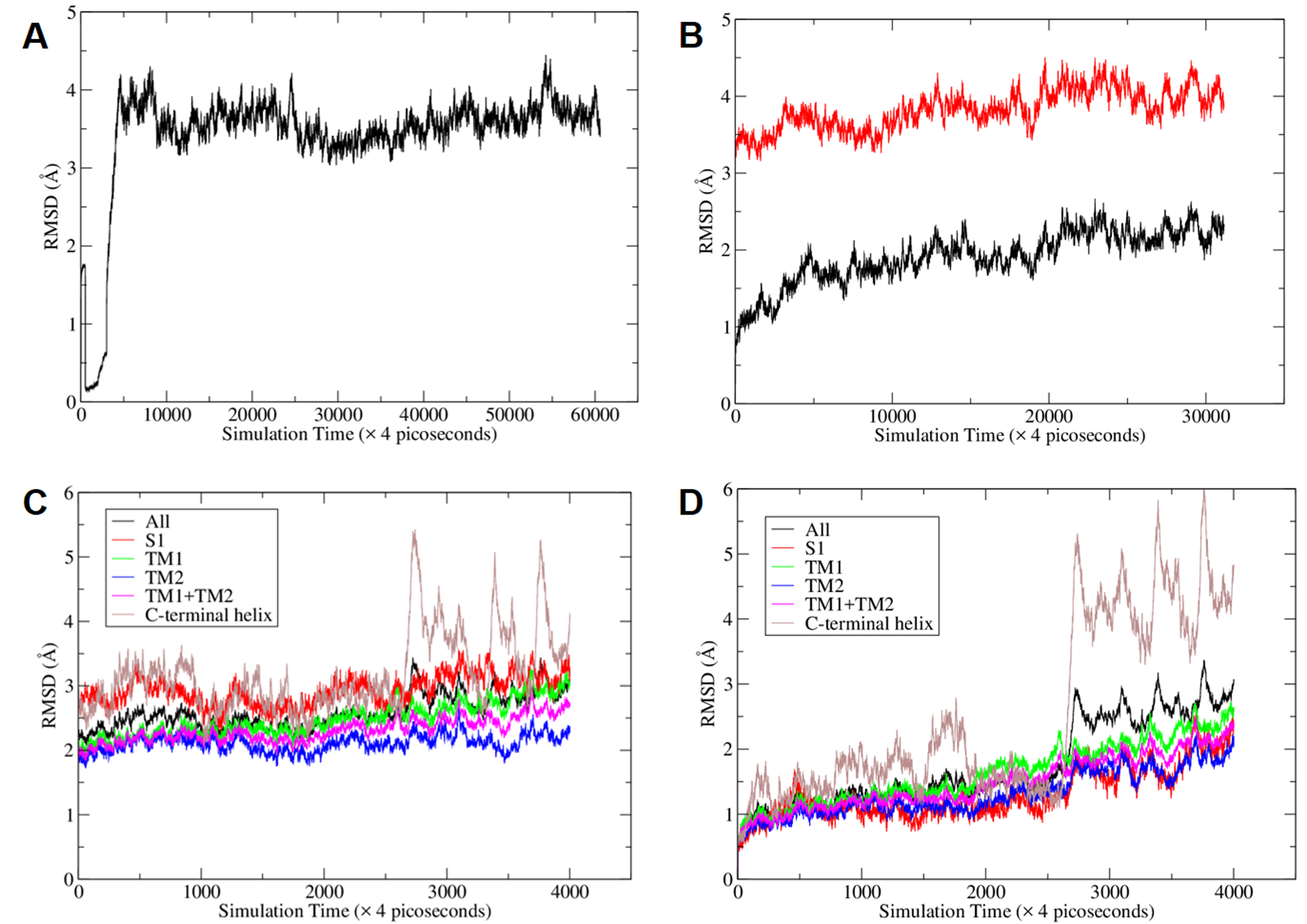

Supplement: S13 Fig — (A) ligand-free MD simulation, the reference structure for RMSD calculation is the crystal structure of Mt-MscL (PDB Code 2OAR). (B) MD simulation of DHS passing through the MscL channel without EEF applied. Red curve represents the RMSDs with the crystal structure of Mt-MscL as the reference, while black curve represents the RMSDs with the last snapshot of the ligand-free MD simulation as the reference. (C) MD simulation of DHS passing through the MscL channel with an EEF strength of 0.2 volt/Å applied to the DHS. Least-squares fitting with the last snapshot of the ligand-free MD simulation of Ec-MscL as the reference structure was first performed for all the α-carbons of Ec-MscL, and then the RMSDs of secondary structure domains (TM1, TM2, TM1+TM2, S1, and C-terminal helix) were calculated directly without fitting. (D) Similar to (c) except that the reference structure in least-squares fitting is the last snapshot of Ec-MscL/DHS MD simulation without EEF being applied. (TIF) [file pbio.1002473.s014.tif]
